# J-type assembled Pt(IV)-coordinated carbon dots for near-infrared light-triggered pyroptosis

**DOI:** 10.1038/s41377-025-01834-w

**Published:** 2025-04-15

**Authors:** Dongbo Guo, Yijie Hou, Qin Xu, Bingzhe Wang, Tesen Zhang, Quansheng Cheng, Maohua Chen, Linxuan Huang, Guichuan Xing, Songnan Qu

**Affiliations:** 1 State key laboratory of digital medical engineering, Sanya Research Institute of Hainan University, School of Biomedical Engineering, Hainan University, Sanya, 572025 China; 2 Joint Key Laboratory of the Ministry of Education, Institute of Applied Physics and Materials Engineering, University of Macau, Taipa, Macau SAR 999067 China; 3 Dongguan Institute of Clinical Cancer Research, Dongguan Key Laboratory of Precision Diagnosis and Treatment for Tumors, The Tenth Affiliated Hospital of Southern Medical University (Dongguan People’s Hospital), Dongguan, 523109 China

**Keywords:** Biophotonics, Synthesis of graphene, Nanoparticles, Biomedical materials

## Abstract

Near-infrared (NIR) light-triggered pyroptosis based on biocompatible Pt(IV)-coordinated nanomedicine for tumor precision therapy is challenging. Here, we disclose a supramolecular approach to construct a hollow-spherical supra-(carbon dots) (HS-Pt-CDs) via ultrasound-assisted J-type assembly of Pt(IV)-coordinated carbon dots (Pt-CDs). The peculiar assembling behaviors arise from the steric hindrance and lattice distortion in the bowl-like Pt-CDs caused by the coordination of Pt(IV) atoms among the sp^2^ domains, which result in around 240 nm red-shifted absorption bands and promoting charge separation in the NIR region due to strong inter-molecular charge transfer (CT) in HS-Pt-CDs. The results reveal that HS-Pt-CDs exhibit excellent NIR light-activated photocatalytic capacities, involving the release of Pt(II) species, the generation of hydroxyl radicals, and acidification under 690 nm laser irradiation. Combined with the effective cellular uptake and tumor accumulation, HS-Pt-CDs can efficiently trigger cancer cell pyroptosis under 690 nm laser irradiation, resulting in the destruction of the primary tumor and effectively induction of strong immunogenic cell death (ICD), thereby evoking anti-tumor immune responses to suppress distant tumor and prevent cancer metastasis. Taken these merits, an important perspective of Pt(IV)-contained supra-CDs with outstanding NIR-triggered photocatalytic behaviors can be of great significance toward precision tumor phototherapy.

## Abstract (web summary)

This study discloses (**a**) J-type assembly approach to construct a hollow-spherical supra-(carbon dots) with around 240 nm red-shifted absorption bands for near-infrared light-triggered pyroptosis.

## Graphical abstract

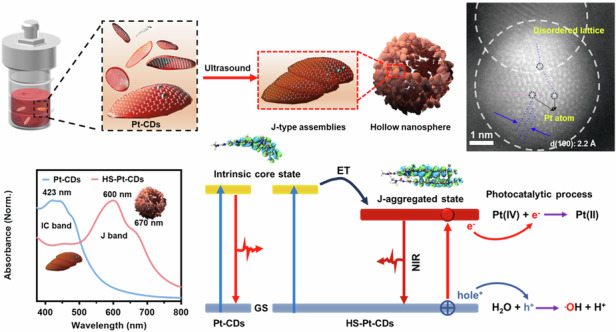

## Introduction

Nanomedicine has garnered growing interest in emerging innovative technologies of cancer immunotherapy, which has steered the field of cancer treatment toward precision medicine^1–3^. Pyroptosis, a form of highly immunogenic cells death (ICD), presents a potential avenue for the development of precision immunotherapy^4–6^. Platinum-based drugs are a category of first-line chemotherapeutic agents approved in clinics. Among the reported pyroptosis-induced agents, photocatalytic Pt(IV)-based nanomedicine, which can precisely release Pt(II) species and generate effective reactive oxygen species (ROS) under certain wavelength light irradiation^7–9^, is of great interest for tumor phototherapy to prevent cancer immune evasion with minimal adverse effects and low drug resistance^10–15^. Long-wavelength photocatalytic Pt(IV)-based nanomedicine with deep tissue penetration and highly accumulation in tumors is prerequisite for clinical translation. Nevertheless, due to the lack of water soluble large π-conjugated nano-scaffolds, current Pt(IV)-contained water-soluble nanomaterials are usually containing small π-conjugated segments, which are mostly activated under ultraviolet-visible wavelength with shallow tissue penetration. Therefore, it is highly desired to develop NIR-activated Pt(IV)-contained nanomedicine for precision tumor phototherapy.

The supramolecular approach provides distinct advantages in constructing fascinating functional nanostructures through regulation of the intra- and inter-molecular photophysical behaviors^16–19^. Harvesting the narrow bandgap excitons via charge transfer (CT) from supramolecular assemblies provides an approach to construct NIR absorption bands^20–22^. J-aggregates with molecules in slip-stacked alignment, favored by the CT process, can realize large red-shifted absorption and emission bands compared to their monodispersed counterparts^23–27^. Carbon dots (CDs), a biocompatible luminescent carbon nanomaterial with sizes less than 10 nm, have garnered widespread attention^28,29^. The employ of nontoxic CDs as building blocks to construct functional supra-architecture has demonstrated to be an effective way to adjust their absorption bands to the NIR region with enhanced photothermal conversion efficiency^30–34^. Furthermore, heteroatom doping and defect engineering would modulate the electronic structures and inter-particle interactions^26,35–39^. Thus, by meticulous regulation of the structures, Pt-atom doping, and assembling behaviors, we aim to develop NIR-light-triggered photocatalytic Pt(IV)-coordinated supra-CDs for precision tumor phototherapy.

**Fig. 1:**
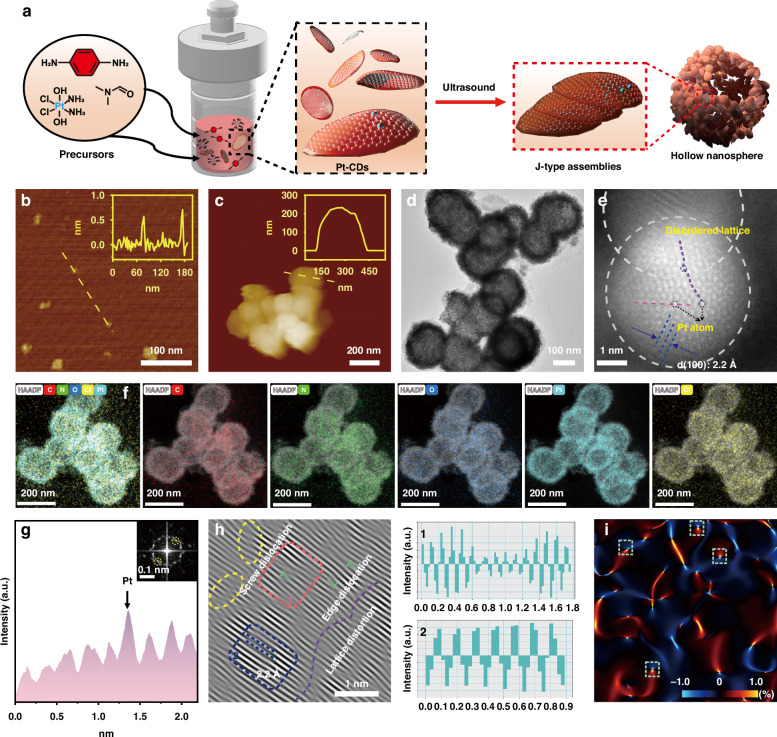
Synthesis process diagram and characterization of J-type aggregates in HS-Pt-CDs. **a** The synthesis and assembly illustration of Pt-CDs to HS-Pt-CDs. AFM images and the height profile analysis (inset) of (**b**) Pt-CDs and (**c**) HS-Pt-CDs. **d** TEM and (**e**) representative AC-HAADF-STEM images of the HS-Pt-CDs. Black circles represent Pt atom. **f** EDX elements mapping images of HS-Pt-CDs. **g** Intensity profile of the atoms dash line in regions of (**e**) and IFFT pattern of HRTEM (inset). **h** The Moiré pattern of HRTEM. Purple dashed lines represent dislocations, blue dashed lines represent lattice fringes. Line-scanning intensity profile obtained from the area highlighted with (1) orange lines and (2) blue lines in (**h**). **i** Strain fields mapping of (**e**) HAADF imaging by geometric phase analysis (GPA) with horizontal normal strain components ε_xx_. The color scale denotes the strain intensity changes from −1.0% (compressive) to 1.0% (tensile). The area of dashed lines indicates the areas around the dislocations

As a proof of concept, we constructed a supramolecular strategy to achieve NIR-triggered prodrug based on the assemblies of Pt(IV)-coordinated carbon dots (Pt-CDs) (Fig. 1a). The Pt-CDs were bowl-like in shape with considerable steric hindrance and lattice distortions coursed by the coordinated Pt(IV) atoms among the sp^2^ domains. This peculiar structure led to J-type slip-aggregation among Pt-CDs, which can further assemble to hollow nanospheres (HS-Pt-CDs) with sizes of ~160 nm after ultrasound treatment. Due to the strong CT processes among Pt-CDs in J-type aggregates, HS-Pt-CDs exhibit more than 240 nm red-shift absorption bands and superior photocatalytic behaviors in the NIR region compared with the monodispersed Pt-CDs. Furthermore, HS-Pt-CDs can be effectively internalized by cancer cells with non- or low cytotoxicity in the absence of light, and show good accumulation at the tumor sites via intravenous injection. The effective NIR-induced photocatalytic processes in HS-Pt-CDs can efficiently release toxic Pt(II) species, generate sufficient hydroxyl radicals, and induce acidification under 690 nm laser irradiation, which synergistically triggered pyroptosis efficiently. In the EMT6 breast tumor mouse model, NIR-triggered pyroptosis based on HS-Pt-CDs not only killed the primary tumor but also effectively induced strong immunogenic cell death (ICD), evoking an anti-tumor immune response that suppressed distant tumor and prevented cancer metastasis, thereby indicating a promising approach for tumor precision phototherapy.

## Results

Oxoplatin was synthesized according to literatures^40–42^. The Pt(IV)-coordinated carbon dots (Pt-CDs) were prepared from p-phenylendiamine and oxoplatin in N, N’-dimethylformamide (DMF) using the solvothermal method. It was interesting to discover that Pt-CDs can assemble into hollow aggregates (HS-Pt-CDs) in water after ultrasound treatment. The structures and morphologies of the Pt-CDs and HS-Pt-CDs were investigated by atomic force microscopy (AFM) and transmission electron microscopy (TEM) (Fig. 1b–d and Supplementary Figs. S1–S3). In TEM observation, Pt-CDs showed monodispersed ultra-small particles with diameters ranging from 4 to 8 nm (Supplementary Fig. S1). HRTEM image of Pt-CDs showed lattice fringes with 0.22 nm, corresponding to the (100) lattice plane of the graphene-like structure (Fig. 1e and Supplementary Fig. S2). Conversely, AFM results of Pt-CDs showed heights of ~0.6 nm, indicating mono- or bilayered graphene-like plates. In contrast, HS-Pt-CDs exhibited hollow-like spheres with diameters of ~160 nm and heights of ~210 nm in TEM and AFM observations, respectively (Fig. 1c and Supplementary Fig. S3). The size changes from Pt-CDs to HS-Pt-CDs were also demonstrated in dynamic light scattering (DLS) (Supplementary Figs. S5 and S6).

To investigate the formation mechanism of the hollow-spherical architecture, the distribution of Pt element was first examined by spherical aberration-corrected high-angle annular dark-field scanning TEM (HAADF-STEM). The STEM and energy dispersive X-ray spectroscopy (EDX) maps showed that C, N, Cl, O, and Pt elements were evenly distributed throughout the HS-Pt-CDs, suggesting that Pt atoms were uniformly coordinated in Pt-CDs and HS-Pt-CDs (Fig. 1f). As the intensity of Pt element was significantly higher than C, O, N, and Cl elements (Fig. 1g), it was reasonable to assign these high contrast atoms to Pt element at the local disordered lattice sites, suggesting the coordination of Pt atoms in the graphene-like plate. The FFT (fast Fourier transformation) and the inverse FFT (IFFT) analysis were performed in the region of local disordered lattice sites. The diffraction pattern of the selected area contained diffraction spots arranged in a pseudo-hexagonal pattern, indicating a clear graphene-like structure (Fig. 1g inset). The Moiré pattern image showed a lot of point and linear defects^43^, leading to considerable lattice distortions, potentially due to the coordination of Pt atoms in the sp^2^ domains (Fig. 1h). Considering Pt-CDs are mono- or bilayered graphene-like plates, the screw dislocations observed in HS-Pt-CDs indicated lattice mismatching happened among the assemblies of Pt-CDs (Fig. 1h). The strain fields derived from the geometric phase analysis (GPA) in Fig. 1i and supplementary Fig. S7 revealed that each dislocation site extended far enough to intersect, comprising symmetrical compression-tension strain pairs. The findings indicated that point defects and dislocations significantly affected strain, shear, and lattice distortion inside the graphene-like plane, which may be the defect-driven deformation into a bowl-like morphology and further aligned in a slip-stacking way to form hollow-spherical architecture^44–46^.

**Fig. 2:**
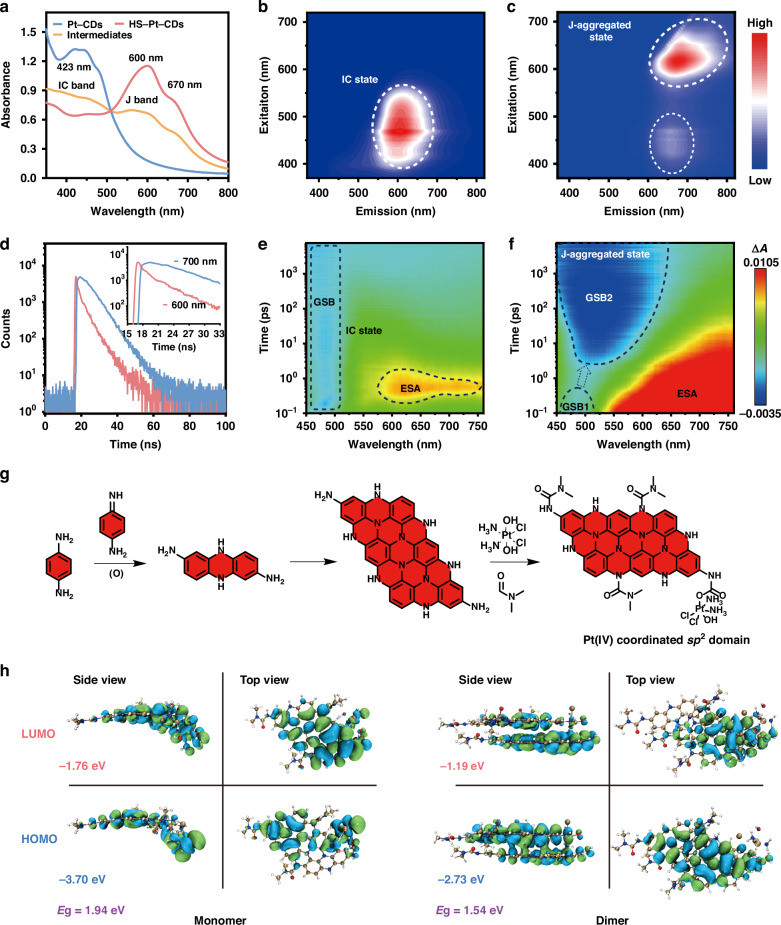
Optical properties of J-type aggregates in HS-Pt-CDs. **a** Absorption spectra of Pt-CDs, intermediates, and HS-Pt-CDs. Excitation-emission maps of (**b**) Pt-CDs and (**c**) HS-Pt-CDs aqueous solutions. **d** Luminescent decay curves of HS-Pt-CDs monitored at 600 nm (red line) and 700 nm (blue line) under 450 nm excitation. 2D pseudo-color maps of the TA spectra of (**e**) Pt-CDs and (**f**) HS-Pt-CDs with a pump wavelength of 400 nm. **g** A proposed formation mechanism of Pt(IV) coordinated sp^2^ domain. Calculated frontier molecular orbitals for (**h**) monomer and dimer of Pt(IV) coordinated sp^2^ domains and their orbital energies in the optimized excited state

**Fig. 3:**
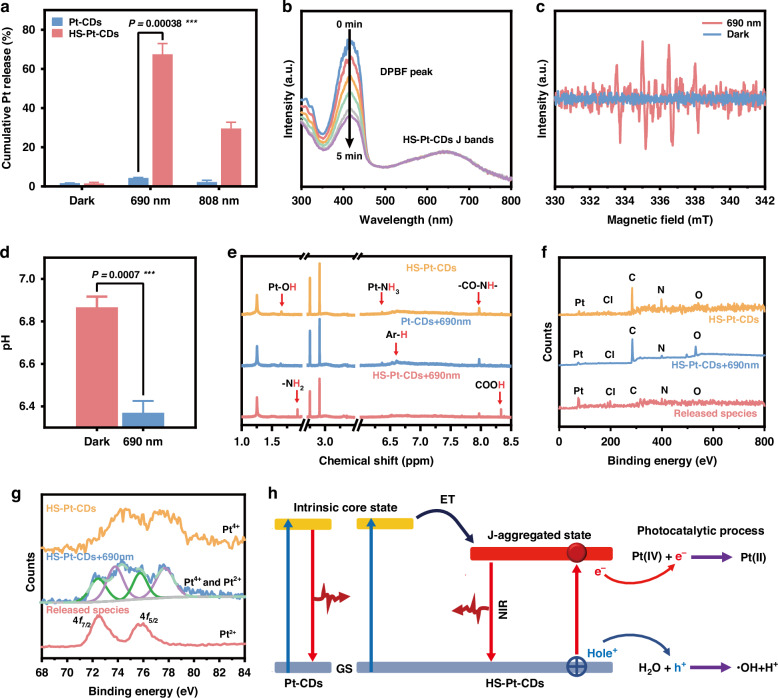
NIR-induced photocatalytic behaviors of J-type aggregates in HS-Pt-CDs. **a** Pt species released profiles of Pt-CDs and HS-Pt-CDs in the dark or after 30 min 690 nm or 808 nm laser irradiation. **b** ROS production of HS-Pt-CDs by measurement of 1,3-Diphenylisobenzofuran (DPBF) peak at 416 nm upon different time 690 nm laser irradiation. **c** EPR spectra of HS-Pt-CDs before and after 690 nm laser irradiation. **d** The pH values of HS-Pt-CDs aqueous solution before and after 30 min 690 nm laser irradiation. **e** ^1^H NMR spectra of HS-Pt-CDs, Pt-CDs+690 nm and HS-Pt-CDs+690 nm in DMSO-*d*_*6*_. **f** XPS survey spectra and (**g**) high-resolution Pt_4f_ spectra of HS-Pt-CDs, HS-Pt-CDs+690 nm, and released species. **h** Illustration of energy level diagrams of the Pt-CDs and HS-Pt-CDs, and a possible NIR light-triggered photocatalytic mechanism for HS-Pt-CDs in aqueous solution. HS-Pt-CDs: 50 μg mL^−1^, NIR: 690 nm or 808 nm laser at 1.0 W cm^−2^

According to literature, *p*-benzene diamine can be condensed into diaminophenazines and subsequently converted to larger oligomers via quinonoid intermediates under solvothermal conditions^47^. Moreover, our previous work demonstrated that Pt(IV) moieties were easily coupled with amino groups via acylation reactions^42^. As shown in Supplementary Table S2, the XPS results showed that Pt-CDs were composed of C, O, N, Pt, and Cl elements with atomic contents of 62.7, 8.4, 24.6, 1.4, and 2.9 mol%, respectively. The ^1^H NMR analysis of Pt-CDs showed the aromatic proton signals ranged from 6.5 to 7.01 ppm and the amide proton signal located at 7.97 ppm (Fig. 3e). Based on the results, the formation of a hypothesized Pt(IV)-coordinated sp^2^ domain in Pt-CDs was depicted in Fig. 2g. A diaminophenazine analogue and larger oligomers were formed by the condensation of p-benzene diamine and quinonoid intermediates. The oxoplatin was attached to the oligomers with spatial steric hindrance to prevent close face-to-face aggregation of the sp^2^ domains. Thus, the Pt(IV) coordinated sp^2^ domains further fused into twisted graphene-like plates to form the bowl-like Pt-CDs.

The optical characteristics for Pt-CDs and HS-Pt-CDs were investigated (Fig. 2a–c). Pt-CDs displayed a principal absorption band peaked at 423 nm. After assembly by ultrasound, the absorption band at 423 nm for monodispersed Pt-CDs diminished, while a longer absorption band peaked at 600 nm and a shoulder absorption centered at 670 nm emerged for HS-Pt-CDs. The excitation and emission maps revealed that the maximum emission center migrated from 600 nm for Pt-CDs to 690 nm for HS-Pt-CDs, while the maximum excitation centers transferred from blue to deep red regions, respectively (Fig. 2b, c). The prominent 240 nm red-shifted absorption bands in HS-Pt-CDs indicated significant electron structural rearrangement during the formation of hollow nanospheres, which resembled the CT band for J-type assemblies. The excitation dynamics of HS-Pt-CDs were investigated by time-correlated single photon counting (TCSPC) (Fig. 2d). The emission decays of HS-Pt-CDs at 600 and 700 nm exhibited asynchronous decay durations. The rising edge of the curve monitored at 700 nm was prolonged to intersect with the range of the decaying edge of the curve monitored at 600 nm, indicating an energy transfer (ET) process from the red emission center to the NIR emission center^48–50^.

The excited-state dynamics of Pt-CDs and HS-Pt-CDs were further investigated by the femtosecond transient absorption (TA) spectroscopy under 400 nm pump wavelength. The representative pseudocolor TA spectra were shown with delay times ranging from 0.1 to 7600 ps. As illustrated in Fig. 2e, the Pt-CDs exhibited a ground state bleaching (GSB) signal in 450–510 nm regions (GSB1), which can be attributed to the intrinsic (IC) state of the graphene-like plate. The excited state absorption (ESA) signal of Pt-CDs exhibited extremely short decay lifetimes of only 0.2 ps (Supplementary Figs. S10 and S13). For HS-Pt-CDs, the GSB1 signal was much weaker and rapidly decayed, while broad and intense GSB signals covered from 450 to 700 nm (GSB2) emerged following the decay of the GSB1 signal. The results revealed the rearranged electronic structures after assembly, with energy transfer (ET) happening from GSB1 to GSB2. Considering the steady-state absorption spectra (Fig. 2a and Supplementary Fig. S12, S15), the GSB2 band can be attributed to the CT band for the J-aggregated state, and significant energy transfer happened from the IC state to the J-aggregated state. These results demonstrated the J-aggregating behavior contributed to the longer wavelength absorption band due to CT processes among the Pt(IV)-coordinated sp^2^ domains in HS-Pt-CDs.

To reveal the assembling behavior of Pt-CDs, we simulated a molecular packing mode of Pt(IV) coordinated sp^2^ domain using density functional theory (DFT) calculations. Geometric model optimization and bandgap calculations of the model in monomer and dimer were performed using the hybrid exchange-correlation density functional (B3LYP) together with 6-31 G(d)+LANL2DZ mixed basis sets in the DFT calculations. In the model, the Pt(IV) coordinated sp^2^ domain was twisted due to the non-coplanarity and steric effect of the coordinated Pt atom (Fig. 2h), whose fusion could result in a bowl-like structure. The considerable steric effect of the coordinated Pt(IV) atom inhibited the face-to-face stacking of the Pt(IV) coordinated sp^2^ domains, resulting in a slip-tendency towards J-type aggregation. The simulated slipping angle between the Pt(IV) coordinated sp^2^ domains was about 41.2° with a distance of ~3.15 Å (Fig. 2h and Supplementary Fig. S16). We proposed that the coordinated Pt(IV) atoms in Pt-CDs caused the lattice distortion and considerable bowl-like architecture, hence facilitating the J-type assembly into hollow-spherical HS-Pt-CDs. The interaction region indicator (IRI) isosurface map showed that the interaction between the Pt-CDs was mainly due to the van der Waals (vdW) interaction within sp^2^ domains (Supplementary Fig. S18). To elucidate the NIR-emitting in HS-Pt-CDs, we calculated bandgaps of the Pt(IV) coordinated sp^2^ domains before and after J-aggregation. The geometrically optimized structures as well as the lowest unoccupied molecular orbital (LUMO) and highest occupied molecular orbital (HOMO) distributions of the model in monomer and dimer in vacuum were presented. In dimer, the HOMO electron densities were distributed on the electron-rich sp^2^ domains, whereas the LUMO electron densities were localized on the electron-deficient Pt(IV) fragment (Fig. 2h), showing a typical CT feature. All the results further indicated a favored long-wavelength photocatalytic process could be achieved in J-type assemblies of Pt(IV) coordinated sp^2^ domains in HS-Pt-CDs.

The photocatalytic behaviors of HS-Pt-CDs were further investigated (Fig. 3a). Pt-CDs and HS-Pt-CDs were stable in the dark, even in the presence of glutathione (Supplementary Fig. S19). Upon irradiation with NIR light, only the HS-Pt-CDs were detected to release Pt-contained species. Nearly 67% of Pt atoms were released from HS-Pt-CDs after 30 min of 690 nm laser exposure. In contrast, negligible Pt-contained species were released from Pt-CDs in the identical laser irradiation. As illustrated in Fig. 3b–d, the HS-Pt-CDs exhibited excellent NIR photo-induced oxidative capability by generating substantial ROS to convert OH^−^ to·OH, as evidenced by the downregulation of DPBF absorption at 416 nm, the separation of 4-line signals with an intensity ratio of 1:2:2:1 in the electron spin resonance (ESR) spectrum, and acidification displayed by reduced pH values.

The mechanism of the light-induced photocatalytic behaviors of HS-Pt-CDs was further investigated by analyzing their structural changes after 690 nm irradiation. The sample of HS-Pt-CDs after 30 min of 690 nm laser irradiation and their released species were collected from the dialysate within and outside the dialysis bag (1000 MWCO), respectively, followed by freeze-drying for further ^1^H NMR, ICP-MS, and XPS analysis. The XPS data indicated the Pt content of HS-Pt-CDs after 690 nm irradiation (HS-Pt-CDs+690 nm) was significantly decreased (Fig. 3f). High-resolution Pt_4f_ spectra of HS-Pt-CDs showed 4f_5/2_ and 4f_7/2_ peaks were located at 77.9 and 74.2 eV, respectively, suggesting the presence of Pt(IV) in HS-Pt-CDs (Fig. 3g). The released species from HS-Pt-CDs after 690 nm irradiation showed obvious Pt, N, and Cl signals with ratios of 1:1.48:1.57, while negligible amounts of O and C elements were detected (Supplementary Table S2). The 4f_5/2_ and 4f_7/2_ Pt signals from the released species were exclusively observed at 75.8 eV and 72.5 eV (Fig. 3g), respectively, indicating the coordinated Pt(IV) atoms in HS-Pt-CDs were reduced and released in the form of Pt(II) coordinated small molecules. In the ^1^H NMR spectrum of HS-Pt-CDs after 690 nm irradiation, the peak at 6.4 ppm for Pt-NH_3_ and peak at 1.75 ppm for Pt-OH were both decreased (Fig. 3e). Simultaneously, the downregulation of -C=O-NH- groups at ~7.97 ppm and upregulation of -NH_2_ at ~1.97 ppm and COOH at ~8.33 ppm in HS-Pt-CDs after 690 nm irradiation further indicated that Pt(II) species were released via photo-induced reduction by cleaving the Pt(IV)-O bonds upon 690 nm irradiation. These results matched well with the downregulated Pt-O signal at ~532.2 eV and an upregulated carboxylic acid signal at ~535.5 eV in the O1s spectra of HS-Pt-CDs after 690 nm irradiation (Supplementary Figs. S21–S23). In contrast, no discernible chemical structural changes of Pt-CDs were detected after 690 nm laser irradiation (Fig. 3e).

Based on the results above, we proposed a possible mechanism for the NIR light-triggered photocatalytic behaviors of HS-Pt-CDs (Fig. 3h). The monodispersed Pt-CDs possessed only an IC state with photoactivity in the blue and green wavelength regions, lacking apparent photocatalytic capacity under 690 nm light. The coupling among Pt-CDs in J-aggregates resulted in the emergence of wide charge transfer bands at long wavelengths, covering from red to NIR wavelength regions. The NIR light can excite CT processes in HS-Pt-CDs, in which NIR photo-excited electrons were captured by the Pt(IV) moiety and happened NIR photo-induced reduction reactions to release Pt(II)-contained small molecules, while the NIR light-generated holes exhibited strong oxidation capacity to produce hydroxyl radicals upon reaction with OH^−^. After gradual consumption with the amount of OH^−^, the proton concentration rose, leading to the acidification of the solution.

**Fig. 4:**
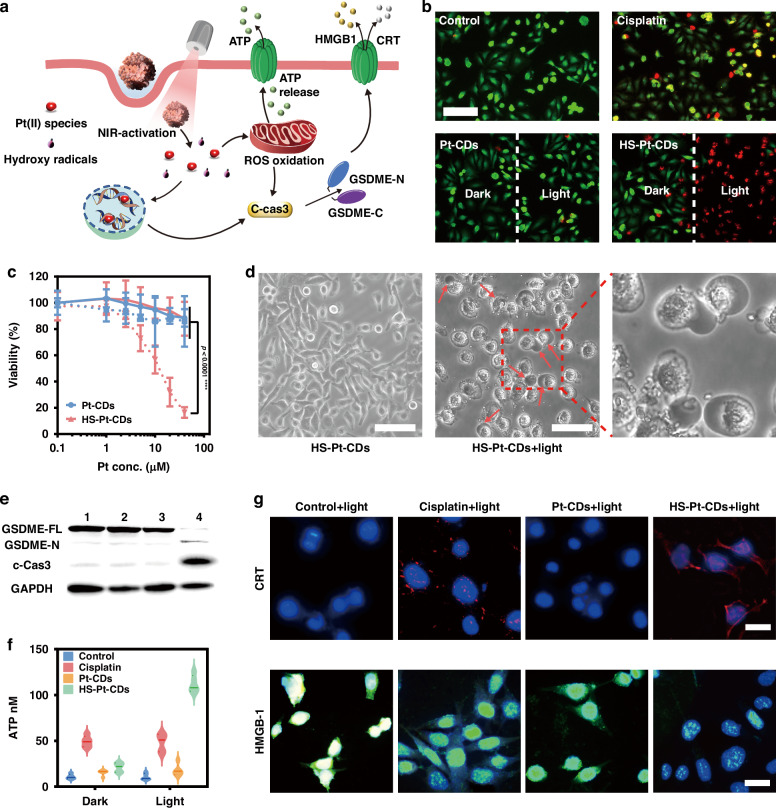
In vitro photocatalytic effect of J-type aggregates in HS-Pt-CDs. **a** Illustration of photocatalytic HS-Pt-CDs triggered pyroptosis under NIR light. **b** Fluorescent images of live/dead staining of EMT6 cells treated with Pt-CDs, and HS-Pt-CDs at 20 μM of Pt with and without 690 nm laser irradiation. Scale bar: 50 μm. **c** Cytotoxicity of EMT6 cells treated with Pt-CDs, and HS-Pt-CDs with or without 690 nm laser irradiation by MTT assay for 48 h (dash lines represent the 690 nm laser irradiated group). Data represent the mean ± SD (*n* = 6). **d** Phase-contrast imaging assays of NIR triggered pyroptosis in EMT6 cells (20 min, 690 nm, 0.5 W cm^−2^). **e** Western blotting demonstrated caspase-3 cleavage of GSDME. c-Cas3, Cleaved Caspase-3; GSDME-FL, full-length GSDME; GSDME-N, the N-terminal cleavage products of GSDME, respectively. 1:blank+light, 2:cisplatin+light, 3:Pt-CDs+light, 4:HS-Pt-CDs+light. **f** ATP secretion from blank-, cisplatin-, Pt-CDs-, and HS-Pt-CDs-treated EMT6 cells at Pt content of 20 μM with or without 690 nm light irradiation. **g** Fluorescence images of CRT expression on EMT6 cells and HMGB1 release from EMT6 cells treated with blank, cisplatin, Pt-CDs, and HS-Pt-CDs at Pt content of with 690 nm laser irradiation. Scale bar: 20 μm. NIR condition: 690 nm laser at 0.5 W cm^−2^, Data presented as mean ± S.D. (*n* = 3)

The cellular uptake of Pt-CDs and HS-Pt-CDs was investigated by monitoring the intracellular red fluorescence signals at 1, 2, 4, 6 h, and 12 h (Supplementary Figs. S24 and S25). The EMT6 cells treated with HS-Pt-CDs exhibited intense red fluorescence signals compared to those treated with Pt-CDs after 6 h of incubation, indicating superior cellular uptake of HS-Pt-CDs over that of Pt-CDs. The internalization mechanism was manipulated by colocation studies with Lyso-Tracker Green. HS-Pt-CDs showed well colocalization with lysosomes (Supplementary Fig. S26), demonstrating cell internalization with endocytosis for HS-Pt-CDs. To reveal the photocatalytic performance, a phase-contrast imaging assay and a live/dead cell viability assay were conducted in EMT6 cells incubated with Pt-CDs and HS-Pt-CDs at Pt content of 20 µM upon 690 nm excitation at 0.5 W cm^−2^. There was no substantial cell death treated with Pt-CDs in the dark and NIR light irradiation (Fig. 4b). However, the EMT6 cells treated with HS-Pt-CDs showed distinct live/death areas between nonirradiated cells with green fluorescence (live) and irradiated cells with red fluorescence (death). Furthermore, an MTT assay was employed to investigate the NIR-triggered cell death with various dosages of Pt-CDs and HS-Pt-CDs (Fig. 4c). The cell viability was nearly unchanged following Pt-CDs and HS-Pt-CDs treatment in the dark, or Pt-CDs under 690 nm light irradiation, even up to the Pt content of 40 μM. In contrast, HS-Pt-CDs showed considerable cytotoxicity exposure to NIR light.

Considering the photo-induced ROS could trigger pyroptosis through the N-terminal domains of gasdermin E (N-GSDME) cleaved by inflammatory caspase-3 and subsequently cause the pore formation in membranes^13,51–53^, the cell morphology observation and the expressions of cleave-caspase-3 (c-Cas3) and GSDME were conducted (Fig. 4a). The EMT6 cells treated with HS-Pt-CDs following 690 nm irradiation (HS-Pt-CDs+light) exhibited considerable pyroptotic cell death behavior, characterized by the swollen and huge bubbles from the plasma membrane (Fig. 4d and Supplementary Fig. S27, S28). In the HS-Pt-CDs+light group, c-Cas3 and N-GSDME were overexpressed (Fig. 4e), whereas other groups showed no apparent expression of the N-GSDME, displaying that the NIR photocatalytic HS-Pt-CDs effectively triggered pyroptosis through the c-Cas3-GSDME pathway.

Then, the ICD biomarkers, calreticulin (CRT), high mobility group box 1 (HMGB1), and ATP expression in EMT6 cells were tested to elucidate the immunogenic form of the photocatalytically triggered pyroptosis. As shown in Fig. 4f, EMT6 cells in the HS-Pt-CDs+light group produced much more ATP secretion than other groups. The expressions of CRT and HMGB1 validated by immunofluorescence staining revealed a robust red signal of CRT on the membrane surface and a diminishing green signal of HMGB1 in the nucleus in the HS-Pt-CDs+light group compared to other groups (Fig. 4g and Supplementary Fig. S29, S30). All the results demonstrated that NIR-triggered cancer cell pyroptosis based on HS-Pt-CDs exhibited a highly immunogenic form of cell death, which was essential for eliciting an effective anti-tumor immunity of the whole body.

**Fig. 5:**
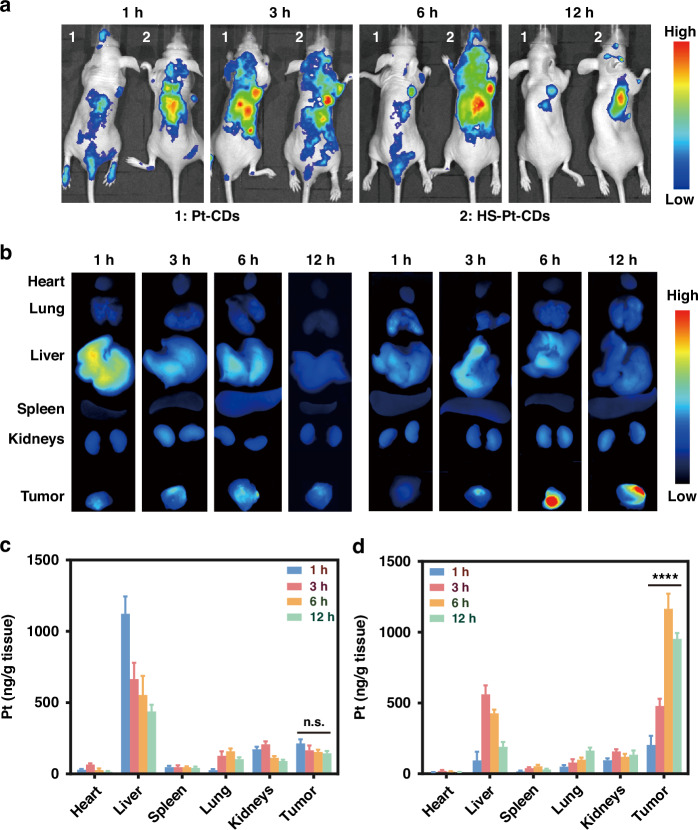
In vivo accumulation of J-type aggregates in HS-Pt-CDs. **a** Live animal fluorescent images of (1) Pt-CDs and (2) HS-Pt-CDs in EMT6 tumor-bearing mice at 1 h, 3 h, 6 h, and 12 h after intravenous injection. **b** Ex vivo fluorescence images of the organs and tumors collected from the executed mice at various time points following Pt-CDs and HS-Pt-CDs intravenous injection. Pt biodistribution in the EMT6 tumor-bearing mice treated with (**c**) Pt-CDs and (**d**) HS-Pt-CDs in the Pt dose of 3.0 mg kg^−1^ (*n* = 3)

The EMT6 tumor-bearing BABL/c mice were used as an animal model to investigate in vivo biodistribution through ex vivo and in vivo imaging. As demonstrated in Fig. 5a, Pt-CDs weakly accumulated at the tumor site within 3 h of intravenous administration and were eliminated within 6 h. Probably due to EPR effect with the increased particle sizes around ~160 nm^54^, HS-Pt-CDs exhibited higher accumulation at the tumor site via intravenous administration within 6 h. Even after 12 h of injection, clear fluorescence can also be observed at the tumor site. For the tissue distribution, the mice were sacrificed at 1, 3, 6, and 12 h after injections of Pt-CDs and HS-Pt-CDs, respectively. In accordance with in vivo imaging, the highest fluorescent signal at the tumor site was found in mice treated with HS-Pt-CDs after 6 h, while the fluorescence signal was not distinctly detectable for other organs (Fig. 5b). The Pt accumulations in organs at 1 h, 3 h, 6 h, and 12 h post-injection was performed by ICP-MS. For the HS-Pt-CDs-treated group, substantial Pt accumulation occurred in the tumor and reached its zenith at 6 h post-injection, which was consistent with in vivo and ex vivo imaging observations. Conversely, only little quantities of Pt-CDs were able to concentrate at the tumor site through blood circulation, whereas most of the Pt was accumulated in the liver within 1 h for the Pt-CDs group (Fig. 5c, d). These findings demonstrated that the assembly of Pt-CDs into enlarged HS-Pt-CDs could enhance their tumor accumulation, which can be prerequisite to exert robust tumor photocatalytic therapy in vivo.

**Fig. 6:**
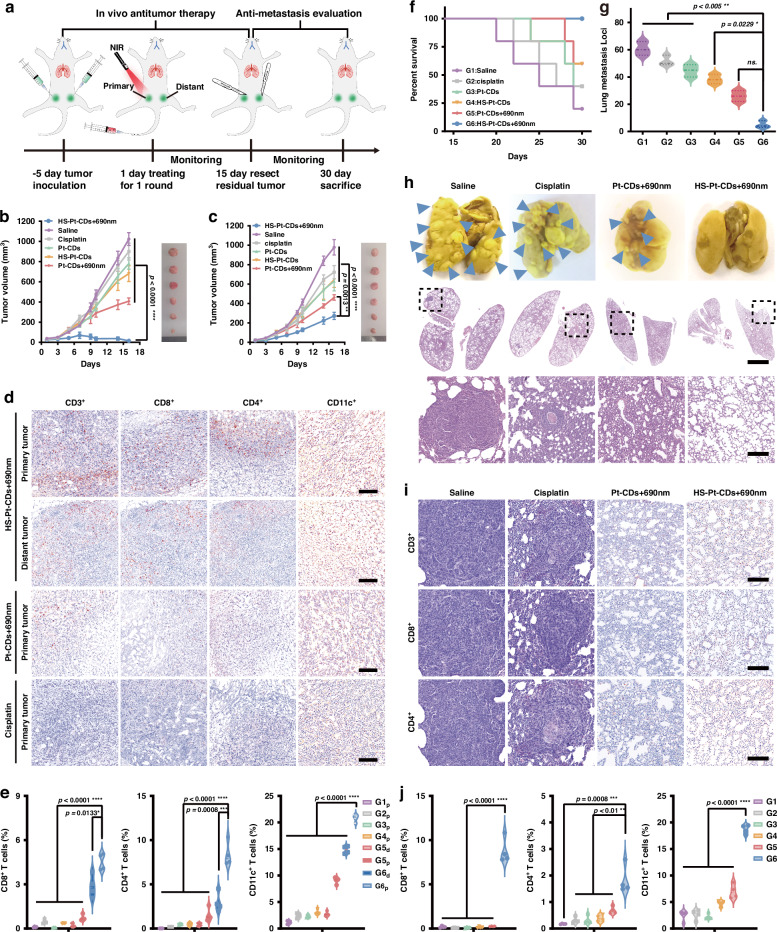
In vivo anti-tumor and lung metastasis effect of photocatalytic HS-Pt-CDs for EMT6 breast tumor. **a** NIR photocatalytic therapy schedule. **b** Primary and (**c**) distant tumor growth curves for BALB/c mice by intravenous injection with one time Pt coordinated drugs treatment (at an equivalent Pt dose of 3.0 mg kg^−1^): G6: HS-Pt-CDs + 690 nm; G5: Pt-CDs + 690 nm; G4: HS-Pt-CDs; G3: Pt-CDs; G2: cisplatin; and G1: saline. **d** Representative immunohistochemical (IHC) images of tumors following a single treatment cycle. Scale bar: 50 μm. **e** Quantitative analysis of CD8^+^, CD4^+^ T cells and CD11c^+^ DCs in primary tumors (G1_p_, G2_p_, G3_p_, G4_p_, G5_p_, G6_p_) and distant tumors (G5_d_, G6_d_) from EMT-6 tumor-bearing mice. **f** Survival curves of the mice following various treatments. **g** Counting of metastatic lung nodules in mice following various treatments. **h** Digital images of lungs stained by H&E and fixed with Bouin’s liquid. The blue triangles denote metastatic nodules. **i** Representative immunohistochemical (IHC) images in lung sections of mice treated with saline, cisplatin, Pt-CDs + 690 nm, and HS-Pt-CDs + 690 nm. **j** Quantitative analysis of CD8^+^, CD4^+^ T cells and CD11c^+^ DCs in lungs. Data presented as mean ± S.D. *p*-values are calculated using two-tailed Student’s *t*-test and two-way ANOVA test, **p < 0.01, ****p < 0.0001)

Effective tumor therapy with one-round treatment is highly desired for overcoming drug resistance^42^. Encouraged by in vitro pyroptosis and ICD induction, one round of NIR-triggered tumor therapy was carried out in EMT6 bilateral tumors of BALB/c mice, as shown in Fig. 6a. EMT6 cells were implanted into each fourth mammary fat pad of healthy female BALB/c mice to create orthotopic bilateral tumors. The right tumor was designated as the distant tumor, while the left tumor was classified as the primary tumor. Following the primary tumor volume reaching ~50 mm^3^, these mice were administered treatment (drugs were injected intravenously with 3.0 mg Pt kg^−1^) and randomly allocated to 6 groups: (G1) saline; (G2) cisplatin; (G3) Pt-CDs; (G4) Pt-CDs followed by NIR light (Pt-CD+690 nm); (G5) HS-Pt-CDs; and (G6) HS-Pt-CDs by NIR light (HS-Pt-CDs+690 nm). Six hours after injection, the primary tumors were exposed to a 690 nm laser (1.0 W cm^−2^, 20 min), whereas the distant tumors remained unlit. The tumor volume increased quickly in G1–G5 groups (Fig. 6b). By comparison, HS-Pt-CDs+690 nm had the potential to provide a strong anti-tumor effect, markedly suppressing primary tumor growth while causing negligible changes in body weights (Supplementary Figs. S31 and S32). In the HS-Pt-CDs+690 nm group, the distant tumors were also suppressed, indicating an anti-tumor immune response was evoked against the death of primary tumor cells (Fig. 6c and Supplementary Fig. S33).

On day 15, each mouse’s tumor was weighed and dissected. Compared to the other groups, the HS-Pt-CDs+690 nm group (G6) showed almost imperceptible primary tumors and markedly reduced distant tumors (Fig. 6b, c). The H&E staining of both the primary and distant tumors in G6 showed a significant area of cell death, accompanied with losses of membrane integrity and nuclear shrinkage (Supplementary Fig. S34). In contrast, negligible cellular death was examined in the primary tumors of G1-G5 mice. The upregulate expressions of cleaved caspase-3 levels and downregulations of ki67 expressions of tumors also revealed considerable cellular death of the primary tumors in G6 (Supplementary Figs. S35 and S36). Thus, it can be concluded that the administration of HS-Pt-CDs+690 nm can both eradicate the primary tumor and suppress the distant tumor.

We then investigated the anti-tumor immune responses by evaluating the expression levels of CD3^+^, CD4^+^, and CD8^+^ T cells, as well as DC maturation (CD11c^+^, CD86, and CD80) in tumors (Fig. 6d and Supplementary Figs. S37–S40). The HS-Pt-CDs+690 nm group showed markedly elevated levels of CD11c^+^ and CD86 DCs, as well as CD4^+^ and CD8^+^ T cells in primary tumors, suggesting photocatalytic therapy can promote DC maturation and facilitate a substantial intratumoral cytotoxic T lymphocyte and helper T cells. For instance, HS-Pt-CDs+690 nm treatment recruited 8.4% and 4.5% of intratumoral infiltration of the CD4^+^ and CD8^+^ T cells, respectively, representing 44-fold and 10-fold increase compared to cisplatin, respectively (Fig. 6e). Meanwhile, the distant tumors in the HS-Pt-CDs+690 nm group also had high levels of CD4^+^ and CD8^+^ expression, measuring 15.6-fold and 6.4-fold higher than those of the Pt-CDs+690 nm group. On the contrary, the tumors of the mice in G1–G5 showed negligible intratumoral infiltration of CD3^+^, CD4^+^, and CD8^+^ T cells. This suggested that the ICD triggered by HS-Pt-CDs+690 nm in the primary tumors elicited a strong anti-tumor immune response that evoked an abscopal anti-tumor effect due to this efficient one-round tumor photocatalytic therapy.

Preventing lung metastasis remains a challenge in cancer therapy. Following the proceeding allograft orthotopic bilateral EMT6 tumor mouse trials, all tumors in all groups were excised on day 15, and the survival curve was further constructed. After day 30, all mice in the HS-Pt-CDs+690 nm group survived, whereas other groups died continuously (Fig. 6f). On day 30, the mice were slaughtered and their pulmonary tissues were harvested. The lung metastatic nodules were subjected to staining, enumeration, and photography. As shown in Fig. 6h and Supplementary Fig. S41, there were no apparent lung metastases in the HS-Pt-CDs+690 nm group, showing that pulmonary metastasis was successfully averted following one-round NIR treatment with HS-Pt-CDs on the primary tumor. In contrast, apparent lung metastases (blue arrowheads) were observed in G1-G5. The data showed that the Pt-CDs+690 nm and HS-Pt-CDs + 690 nm treated groups had approximately 57% and 91% less numbers of lung nodules, respectively, in relative to the saline group (Fig. 6g). The H&E results revealed extensive tumor lesions in the lungs, whereas negligible metastatic lesions in the lungs can be observed for the HS-Pt-CDs+690 nm group (Fig. 6h and Supplementary Fig. S41). Then, T-cells and DCs populations in lung tissues were examined to validate the systemic immune response effect. Compared with cisplatin treatment, tumor-infiltrating CD4^+^, CD8^+^ T cells, and CD11c^+^ DCs in the lung tissues rose significantly in HS-Pt-CDs+690 nm treated groups with proportions of 1.8%, 8.8%, and 18.7%, respectively (Fig. 6i, j, and Supplementary Figs. S42, S43). Therefore, the aroused activation of cytotoxic T lymphocytes after HS-Pt-CDs+690 nm treatment has the capability of restrain lung metastasis of breast cancer.

## Discussion

**Scheme 1:**
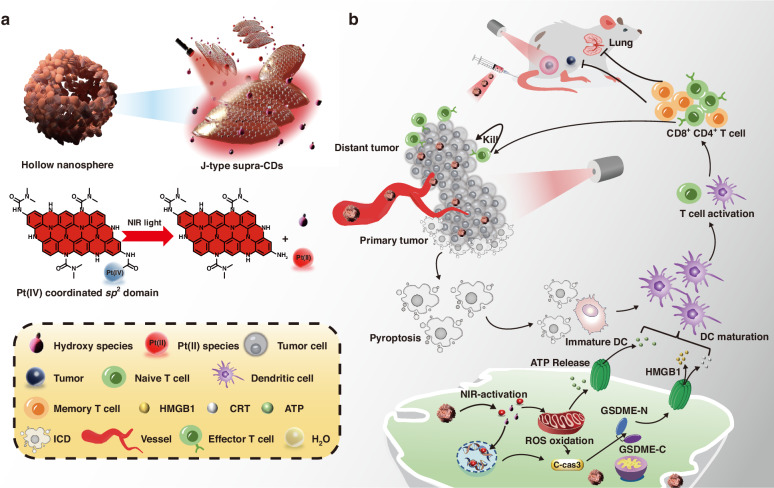
Schematic diagram of photocatalytic behaviors in J-type HS-Pt-CDs. **a** Structures and photocatalytic behaviors of J-type aggregates in HS-Pt-CDs under NIR irradiation. **b** Schematic illustration of photocatalytic HS-Pt-CDs that promote the NIR-triggering Pt(II) species release and ROS to trigger tumor pyroptosis to prevent cancer metastasis

In summary, we reported a novel supramolecular engineering strategy to construct Pt(IV)-coordinated supra-(carbon dots) (HS-Pt-CDs) with NIR-activated photocatalytic capacity to trigger tumor pyroptosis for precision tumor phototherapy (Scheme 1). The coordination of Pt(IV) in sp^2^ domains caused steric hindrance and lattice distortions, inducing the formation of defect-driven bowl-like Pt-CDs, which can further assemble into the hollow-spherical HS-Pt-CDs in J-type slip-aggregation way upon ultrasound treatment. The strong inter-molecular CT processes in HS-Pt-CDs with around 240 nm red-shifted absorption bands promoted charge separation in the NIR light region, efficiently releasing toxic Pt (II) species, generating sufficient hydroxyl radicals, and inducing acidification under 690 nm laser irradiation. Due to the superior biocompatibility and increased particle size, HS-Pt-CDs can be effectively uptaken by cancer cells with non- or minimal cytotoxicity in the absence of light. They showed enhanced accumulation at the tumor sites via intravenous injection, which can effectively trigger pyroptosis under NIR light irradiation. In the EMT6 breast cancer mouse model, after one-round treatment, the NIR-triggered pyroptosis based on HS-Pt-CDs not only eradicated the primary tumor but also effective induced an adequate ICD to evoke anti-tumor immune response to suppress distant tumor and prevent cancer metastasis. We prospect that the NIR light-triggered pyroptosis based on biocompatible Pt(IV)-coordinated supra-CDs has great potentials as a promising nanomedicine for precision phototherapy in clinical oncology.

## Materials and methods

### Preparation and characterization of Pt-CDs and HS-Pt-CDs via Ultrasound

In this work, the Pt(IV)-coordinated CDs were synthesized using the protocol outlined in our earlier research^42^. After dissolving 15 mg of p-phenylendiamine and 15 mg of oxoplatin in 30 mL DMF, the mixture was heated to 200 °C for 6 h in a reaction autoclave using solvothermal conditions, and it was then cooled to room temperature. Using a 1000 MWCO dialysis membrane, red solution was obtained and dialyzed against DI water for a few days before being freeze dried for subsequent studies. Pt-CDs with dark powder were ultimately produced. Next, 5 ml of H_2_O/DMF solution were mixed with 0.5 milligrams of Pt-CDs. For five minutes, the solution was subjected to an ultrasound (XU-PS-20A, 160 W) to create hollow-like nanospheres (HS-Pt-CDs). Transmission electron microscopy (TEM) images, HAADF-STEM images, SAED patterns and Moiré pattern images were collected on a thermoscientific Talos F200X G2 electron microscope (an accelerating voltage of 200 kV) and an aberration-corrected a thermoscientific Talos Spectra 300 S/TEM (accelerating voltage of 60 kV).

## Supplementary information

Supplementary Information

## Data Availability

The data used to generate the plots and results in this paper are available from the corresponding author upon reasonable request.
