# Supplementary material for: J-type assembled Pt(IV)-coordinated carbon dots for near-infrared light-triggered pyroptosis
Source: Light Sci Appl. 2025 Apr 15;14:163. doi: 10.1038/s41377-025-01834-w (PMC12000447; doi:10.1038/s41377-025-01834-w)
Supplement: Supplementary file 1 — Supplementary Information [file 41377_2025_1834_MOESM1_ESM.pdf]

Supplementary Information for

J-Type Assembled Pt(IV)-Coordinated Carbon Dots for Near-Infrared Light-Triggered Pyroptosis

Dongbo Guo[1, 2, #, *], Yijie Hou[1, #], Qin Xu[1], Bingzhe Wang[2], Tesen Zhang[2], Quansheng Cheng[2], Maohua Chen[1], Linxuan Huang[3], Guichuan Xing[2], and Songnan Qu[2, *]

[1] State key laboratory of digital medical engineering, Sanya Research Institute of Hainan University, School of Biomedical Engineering, Hainan University, Sanya, China
[2] Joint Key Laboratory of the Ministry of Education, Institute of Applied Physics and Materials Engineering, University of Macau, Taipa, Macau SAR, China
[3] Dongguan Institute of Clinical Cancer Research, Dongguan Key Laboratory of Precision Diagnosis and Treatment for Tumors, The Tenth Affiliated Hospital of Southern Medical University (Dongguan People's Hospital), Dongguan, China

[#] These authors contributed equally: Dongbo Guo, Yijie Hou.
*Correspondence:
Dongbo Guo (dongboguo@hainanu.edu.cn) or Songnan Qu (songnanqu@um.edu.mo);

Table of Contents

Supplemental experimental procedures

Figures S1-S44
Tables S1-S2

References

Methods

Materials and Characterization

All reagents had at least analytical grade purity and were used as received without further purification. Cisplatin, hydrogen peroxide, p-phenylendiamine, and 3-(4,5-dimethylthiazol-2-yl)-2,5-diphenylte-trazolium bromide (MTT) were purchased from Aladdin. Ferrostatin-1, propidium iodide, Z-VAD, and N-acetylcysteine were purchased from MCE (MedChemExpress). Live/Dead Cell Double Staining Kit, sodium hydrosulfite, anhydrous dimethylformamide (DMF), lysozyme, pepsin, trypsin, and other chemicals were purchased from Sigma-Aldrich. LysoTracker Green, ATP assay kit and 2',7'-bis-(2-carboxyethyl)-5-(and-6)-carboxyfluorescein acetoxymethyl ester (BCECF-AM) was purchased from Beyotime. HMGB1 Polyclonal Antibody, Calreticulin Polyclonal Antibody, and Donkey anti-Rabbit IgG (H+L) Highly Cross-Adsorbed Secondary Antibody, Alexa Fluor™ Plus 488 were purchased from ThermoFisher Scientific. Cleaved Caspase-3 (Asp175) Antibody, CD3ε mAb (E4T1B), CD4 Rabbit mAb (D7D2Z), CD8α (D8A8Y) Rabbit mAb, CD11c (D3V1E) Rabbit mAb, and anti-phospho-H2A.X (Ser139) were purchased from Cell Signaling Technology (CST). Anti-cleve-caspase-3 antibody and anti-ki67 antibody was purchased from Abcam.

UV-vis absorption spectra measured on a Thermo Fisher Evolution 220 spectrophotometer. Visible and near-infrared photoluminescence (PL) spectra were obtained using fluorescence Spectrometer (Edinburgh FS1000). Fourier transform variation infrared spectroscopy (Frontier). AFM measurements were performed using a BRUKER ICON instrument. Raman spectra were acquired using a Horiba LABHRev-UV Raman spectrometer. ^{1}H NMR was measured by a Bruke Advance 400 Spectrometer. The electron paramagnetic resonance (EPR) spectroscopy measurements were recorded on a Magnettech ESR5000 (Bruker). Femtosecond TA spectra of various samples were collected using the Ultrafast Systems HELIOS TA spectrometer. The laser source used was the Coherent Astrella-1 K-F ultrafast Ti:sapphire amplifier (1 kHz, <100 fs). The 400-nm pump pulse was generated by a Light Conversion TOPAS-C optical parametric amplifier. Fluorescence images of cells and mice were obtained using a fully automated inverted differential interference microscope (Leica DMi8) and a bioimaging system (AniView Phoenix 600SE), respectively. The Pt biodistribution amount of Pt-CDs and HS-Pt-CDs were obtained using Thermo Scientific ICP-MSiCAP™ TQ iCAP 7000. The mean particle diameter the Pt-CDs and HS-Pt-CDs were measured by dynamic light scattering (DLS, Malvern Zetasizer Pro). The calculations were conducted in part of the high-performance computing cluster (HPCC) supported by the Information and Communication Technology Office (ICTO) of the University of Macau. All the animal experimental procedures were conducted according to the guidelines of the Regional Ethics Committee for Anima Experiments and the Care Regulations approved by the Institutional Animal Care and Use Committee of the Hainan University (protocol no. HNUAUCC-2023-00004).

NIR-triggered Pt-CDs and HS-Pt-CDs

0.5 mg mL^{-1} of Pt-CDs and HS-Pt-CDs was performed in dialysis bags using an H_2O solution. The fluorescence (FL) and ultraviolet-visible (UV-vis) absorption of Pt-CDs was performed by Edinburgh FS1000 spectrophotometer and Thermo Fisher Evolution 220 spectrophotometer. A 690 nm light was utilized to irradiate at a power of 1.0 W cm^{-2} for 30 min. The dialysis bags' exterior and inside solutions were separated and lyophilized, respectively. The solid powders were analyzed using 1H NMR (Bruke Advance 400) and XPS (Axis Supra, Shimadzu). Glass flasks containing 0.1 M PBS were used to hold the Pt-CDs and HS-Pt-CDs. The Pt-CDs and HS-Pt-CDs were

put into glass bottles with 0.1 M PBS. The Pt release of Pt-CDs and HS-Pt-CDs were acquired from the outer dialysis bags using a Thermo Scientific ICP-MSiCAP™ TQ iCAP 7000.

ESR measurement

The NIR light source was a NIR laser (690 nm, LR-RSP-690/1). DMPO was used as the capture agent to detect ·OH by EPR. The Pt-CDs and HS-Pt-CDs (50 μg mL^{-1}) with DMPO (0.09 mol L^{-1}) were set up in capillary tubes, and the ESR was tested before and after NIR irradiation.

Geometric phase analysis

Geometric phase analysis (GPA) is a technique for measuring strain fields obtained from high-resolution electron microscope images, which is investigated using the strain++ software based on HRTEM images.[1] It elucidates the variation of spatial frequency components (lattice fringes) over the image's field of view.

Theoretical simulation

All theoretical simulations were performed on the Gaussian 16 suite. Density functional theory (DFT) computations were conducted on the built molecular model, utilizing the B3LYP functional in combination with the D3BJ dispersion correction. Geometric model optimization and bandgap calculations of the model in monomer and dimer were performed using the hybrid exchange-correlation density functional (B3LYP) and 6-31G(d)+ LANL2DZ mixed basis sets in the DFT calculations. The Multiwfn and VMD software packages were used to determine the energy level structure of each molecule and dimer and to process the visualization of HOMO and LUMO. Interaction region indicator (IRI) was calculated with Multiwfn 3.8(dev).[2] All structures and isosurfaces images were visualized by VMD 1.9.3.[3]

Imaging of pyroptotic cells

Briefly, 6 well plates were seeded with EMT6 cells and maintained for 24 hours. Following a 4-hour incubation period in 20 μM of Pt, the cells in each group were treated as follows: Pt-CDs, HS-Pt-CDs, Pt-CDs+NIR, and HS-Pt-CDs+NIR treatments. An NIR laser operating at 0.5 W cm^{-2} was used for 20-40 minutes. Phase-contrast images were acquired using a confocal laser scanning microscope (Olympus FV3000, Japan) following the treatment. A more detailed explanation of the experiment used to image the swelling bubbles is as follows: the cells were carefully put in the incubator following the treatment. Every 20 minutes after that, they were examined under a microscope. Upon the identification of morphological abnormalities, the cells were immediately photographed and subsequently washed with phosphate-buffered saline warmed to 37 °C.

Extracellular pH regulation of Pt-CDs and HS-Pt-CDs.

Using 35-mm^2 dishes, extracellular pH was measured using a microprobe-based pH system (Micro pH Electrode, Thermo Fisher). The electrode was calibrated with pH standards and placed in the media of the 35-mm^2 dishes containing the cells. pH measurements were made using an Accumet Ab150 pH meter (Fisher Scientific).

Cell lines and culture

EMT6 murine breast cancer cells, and EMT6 murine breast cancer cells were obtained from

Chinese Academy of Sciences Cell Bank (Shanghai, China). EMT6 cells were maintained in Roswell Park Memorial Institute (RPMI) 1640 medium (GIBCO) supplemented with 10% fetal bovine serum (GIBCO), 100 U mL^{-1} penicillin and 100 μg mL^{-1} streptomycin (GIBCO). EMT6 were maintained Dulbecco's modified eagle medium (DMEM) medium (GIBCO) supplemented with 10% fetal bovine serum (GIBCO), 100 U mL^{-1} penicillin and 100 μg mL^{-1} streptomycin (GIBCO). All cells were cultured in a humidified incubator (5% CO_2 at 37 °C).

Cell viability assay (MTT assay)

The cytotoxicity characteristics of Pt-CDs, and HS-Pt-CDs against EMT6 cells were evaluated by the MTT assay (Aladin). In brief, 5000 cells were initially seeded in 96-well plates and allowed to incubate for 12 hours. Subsequently, 200 μL of new medium containing CDs at different concentrations (1.0, 2.0, 5.0, 10.0, 20.0, and 50.0 μM of Pt) was added to the medium. The cells were either left in the dark or exposed to an NIR laser (690 nm, 0.5 W cm^{-2}) following a 4-hour incubation period. Following a 48-hour incubation period, 200 μL of fresh media containing 0.8 mg mL^{-1} of MTT was added to the cells, and they were incubated for an additional three hours at 37°C. The absorbance at 450 nm was recorded using a SpectraMax iD3 Microplate Reader (MolecularDevices) and normalized to the cells subjected to the dark. Each experiment was performed in triplicate for each cell line.

Cell uptake of Pt-CDs and HS-Pt-CDs.

EMT6 cells (1.2 ×10^6 cells/well) were inoculated in 6-well plates. Thereafter, Pt-CDs and HS-Pt-CDs (20 μM of Pt) were added to the medium and incubated for 1, 2, 4, 6, and 12 h incubation. After that, the cells were rinsed 3 times with PBS, and cell nuclei were labeled with DAPI (Beyotime Biotech, China) to identify the CDs location inside the cells. Cellular images were acquired using an Olympus FV1000 confocal laser scanning microscope (Olympus FV3000, Japan) imaging system. The cells were excited at 543 nm.

Live/Dead assay

To evaluate the effect of the Pt-CDs and HS-Pt-CDs on EMT6 cells, 1×10^5 cells were seeded in glass bottom dishes and cultured overnight. Subsequently, the cells were treated with Pt-CDs or HS-Pt-CDs (20 μM of Pt) for 6 h, illuminated to an NIR light for ten minutes, and then stained with propidium iodide (PI) and calcein acetoxymethyl ester (Calcein-AM) for thirty minutes at 37 °C. After washing three times with PBS solution, the cells were observed with a fluorescent microscope (Olympus FV3000 Inverted Microscope) using a 20x objective lens at 470 nm excitation with a 500 nm LP filter.

Subcellular Localization.

EMT6 cells were seeded at a density of 2 × 10^5cells/well in a 24-well plate over glass coverslips and incubated at 37 °C under 5% CO_2 overnight. After being incubated with Pt-CDs and HS-Pt-CDs ([Pt] = 20 μM) for 3 h at 37 °C, the culture medium was carefully aspirated, washed with DPBS three times, and fixed with 1 mL of 4%paraformaldehyde for 15 min, followed by staining with Lyso Tracker Green for additional 0.5 h at 37 °C in dark. After the extracellular fluorescence was quenched, cells were washed twice with PBS and fixed with 4% paraformaldehyde for 30 min. Then, cells were mountedon glass slides and visualized using confocal microscope (Olympus FV3000,

Japan).

ATP release assay

After being seeded into 96-well plates at a density of 1 × 10^4 cells per well, EMT6 cells were incubated overnight. A unique formulation (Pt: 20 μM) was then applied to each well, and after 24 hours of incubation, the wells were exposed to 10 minutes of 0.5 W cm^{-2} NIR laser radiation. Four hours after treatment, the cell supernatants were collected, and an ATP assay kit (Beyotime Biotechnology, S0026) was employed to quantify the ATP levels.

Intracellular Detection of HMGB1 and CRT (Immunofluorescence, IF)

After being implanted at a density of 1×10^5 cells per well in a 24-well plate with coverslips inside, EMT6 cells were incubated overnight. The cells in the different groups were incubated for 4 h in 20 μM of Pt and then subjected to the following treatments: incubated with cisplatin, Pt-CDs, HS-Pt-CDs, Pt-CDs+NIR, and HS-Pt-CDs+NIR. An NIR laser operating at 0.5 W/cm^2 was used for ten minutes. After that, the EMT6 cells were permeabilized with 0.1% Triton X-100 and fixed with 4% paraformaldehyde. The cells were then subjected to treatment with an Alexa Fluor 488 conjugated anti-rabbit secondary antibody (Beyotime Biotechnology, A0423) and anti-HMGB1 (cell signaling, #3935). Secondly, the cells were treated with a Cy3-conjugated anti-rabbit secondary antibody (Beyotime Biotechnology, A0516) and anti-CRT antibodies (cell signaling, #12238). The cells were counterstained with Hoechst 33258, and confocal laser scanning microscopy was utilized to detect the HMGB1 fluorescence and the CRT fluorescence.

Western blot analysis

The level of cleaved Caspase-3, GSDME, and GAPDH protein expression were quantified by western blot analysis. The EMT6 cells were isolated and subjected to a cold PBS wash after treatment. Following an ice-cold incubation period in Beyotime Biotechnology's RIPA lysis reagent buffer (P0013), the cell lysates were subjected to ultrasound and centrifugation (5,000 g, 10 min). A BCA kit was used to measure the protein concentration (Beyotime Biotechnology, P0012). Following their separation using 15% w/v SDS-polyacrylamide gel electrophoresis (BeyoGelTM Plus PAGE, P0465S), the protein samples (30−45 μg) were then transferred to polyvinylidene difluoride membranes. After 20 minutes of room temperature incubation, the membranes were incubated for an additional hour at 4°C with the primary antibody-cleaved Caspase3 (cell signaling, #9661s), GSDME (Abcam, # ab215191), and HRP-conjugated GAPDH (Yeasen, 30203ES50) at a 1:1,000 dilution. The following day, the secondary antibody (Abcam, #ab6789) was incubated on the membrane after it had been three times cleaned with TBST (Beyotime Biotechnology, ST673). The protein expression levels were detected using enhanced chemiluminescence reagents (Thermo fisher, 34580) and photographed using by a Bio-Rad ChemiDoc XRS+ imaging system.

Live Animals Imaging, and Distribution

The EMT6 tumor models of the nude mice were generated by fat pads injection of EMT6 cell in 100 μL (5 × 10^5 cells) into the dorsal area of each EMT6 mouse. Pt-CDs and HS-Pt-CDs (at an equivalent Pt dose of 3.0 mg kg^{-1}) were intravenously injected into mice with EMT6 orthotopic tumors via the tail vein. At different times (n=3 at each point), primary tumors and other organs were collected and subjected to fluorescence imaging using an IVIS Spectrum Imaging System

(Caliper Life Sciences Inc., USA). Mice were euthanized promptly after 1, 3, 6, and 12 hours for in vivo biodistribution. Orthotopic tumors and major organs (heart, liver, spleen, lung, and kidneys) were removed, and the weight of each organ was noted. The Pt content of tissues and tumors were performed by ICP-MS.

In vivo antitumor effect

Balb/c mice were used to establish a mouse orthotopic breast tumor model by injecting 1×10^6 EMT-6 cells subcutaneously into two mammary fat pads. Six days later, the mice (n = 10) were divided into six groups at random and given intravenous injections of cisplatin, Pt-CDs, and HS-Pt-CDs (at an equivalent Pt dose of 3.0 mg/kg). After six hours, the primary tumor was treated with photocatalysis using NIR light (1.0 W cm^{-2}) for ten minutes. Tumor volume (calculated as tumor volume = $L \times W^2/2$, where W represents width and L represents length) and body weight were monitored every two days. In addition, mice were photographed and recorded on Day 15. Mice body weight, mortality, and clinical status were carefully recorded. Tumors were excised following the anesthesia of all animals on Day 15. Five mice of every group continuously examined to assess survival and tumor metastasis. Following sacrifice of the EMT6-bearing mice, the tumor tissues were collected and fixed with 4% paraformaldehyde (PFA). The tumor tissue was subsequently embedded in paraffin, sectioned into 5 μm sections, deparaffinized, and rehydrated. Antigen retrieval was performed using citrate buffer (pH 6.0) at high temperature in a pressure cooker. Goat serum was employed to inhibit nonspecific binding of antibodies. Primary antibodies, including CD11c, CD3ε, CD4, CD8α, ki67, and c-cas3 were employed for immunohistochemical staining. The panoramic DESK digital pathology scanner (3D HISTECH) was employed to scan all the sections.

In vivo anti-metastasis effect

Following the 15th day of the in vivo anticancer trial, tumors were excised after anesthetizing all the animals on Day 15. Five mice of every group were continuously examined to assess survival and tumor metastasis. Following an additional 15 days of observation, all mice were euthanized on day 30 post-initial medication therapy, and different organs and tissues, including the liver, kidney, heart, lung, and tumor, were taken, fixed, and evaluated by histological analysis. The lungs of mice were collected, rinsed with PBS and fixed in Bouin's fixative solution for 24 h, and eventually photographed. The lungs were also embedded in paraffin, sectioned into 5 μm sections, deparaffinized, and rehydrated. Antigen retrieval was performed using citrate buffer (pH 6.0) at high temperature in a pressure cooker. Goat serum was utilized to prevent nonspecific binding of antibodies. Primary antibodies, including CD11c, CD3ε, CD4, and CD8α were employed for immunohistochemical staining. The panoramic DESK digital pathology scanner (3D HISTECH) was utilized to scan all the sections.

Statistical analyses

All experiment results were given as mean ± standard deviation (s.d.) ($n \geq 3$). The software ImageJ v1.5 was used to quantify the immunofluorescence intensity. Statistical calculation of experimental data was analyzed using the two-way ANOVA test (more than three independent groups, GraphPad Prism 9.5) or a two-tailed Student's t-test (two independent groups, GraphPad Prism 9.5) after normality and equal variance tests. Article drawing was performed using Origin 2021 or GraphPad Prism 8.0.1 software.

Table S1. The stable study of HS-Pt-CDs under different days.

Size (nm)	10 Day	20 Day	30 Day
Pt-CDs	7±2	8±3	8±2
HS-Pt-CDs	156±16	168±20	158±25

Table S2. The atomic ratio of C, N, O, Cl, and Pt in HS-Pt-CDs, ir-HS-Pt-CDs, and released species

	C(%)	N(%)	O(%)	Cl(%)	Pt(%)	Pt(wt%)
HS-Pt-CDs	62.7	24.6	8.4	2.9	1.4	16.9
HS-Pt-CDs+NIR	68.6	9.3	19.9	1.5	0.7	9.4
Released species	N/A	34.2	6.5	36.2	23.1	70.7

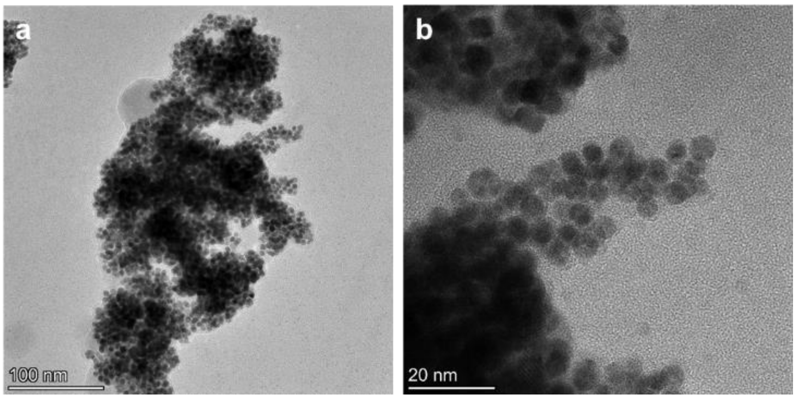

Fig. S1. TEM images of the (a, b) Pt-CDs.

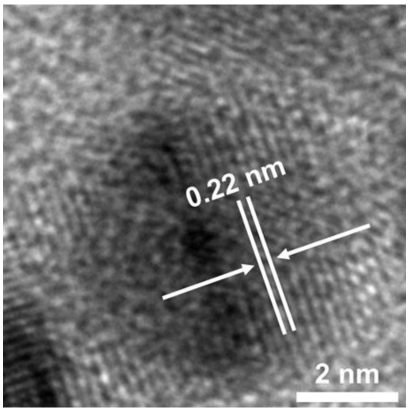

Fig. S2. HRTEM images of the Pt-CDs.

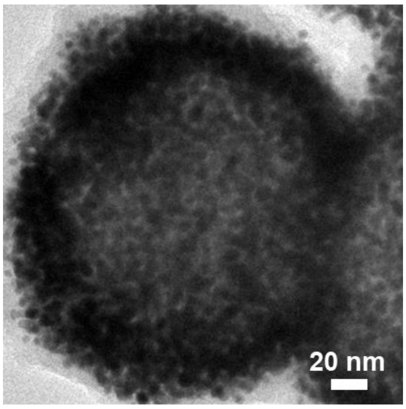

Fig. S3. TEM images of the HS-Pt-CDs.

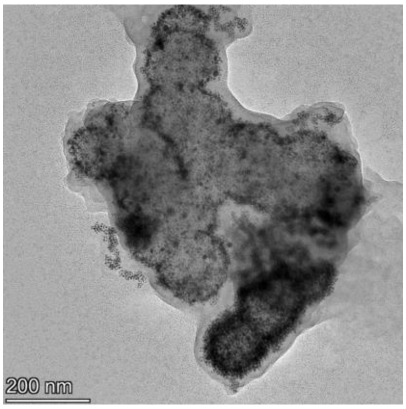

Fig. S4. TEM images of the intermediates under 1 min ultrasound treatment. The monodisperse Pt-CDs undergo spontaneous self-assembly into hollow nanostructures upon ultrasound treatment.

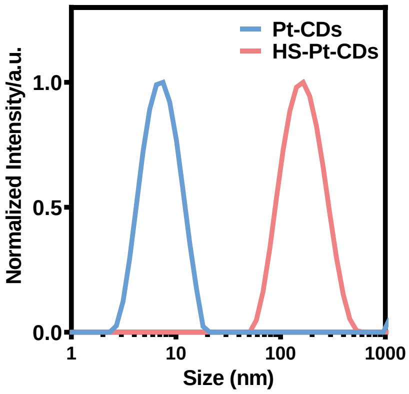

Fig. S5. DLS curves of the Pt-CDs and HS-Pt-CDs.

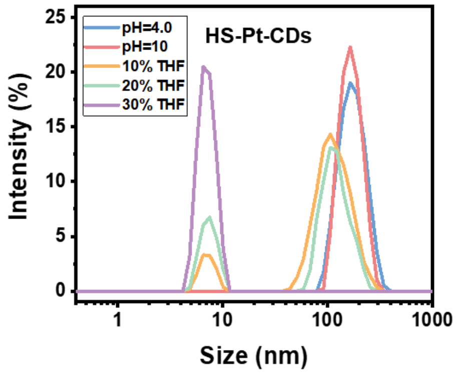

Fig. S6. DLS curves of the HS-Pt-CDs at different pH value and THF. By changing the pH value from 4.0 to 10.0, the size of J-aggregates did not change significantly, indicating that the J-aggregates were stable under the acid-base microenvironment. Then, we further changed the solvent polarity of the medium, as the solvent polarity decreased by THF, the size of J-aggregates rapid decreased, suggesting that disaggregation of J-aggregates was happened.

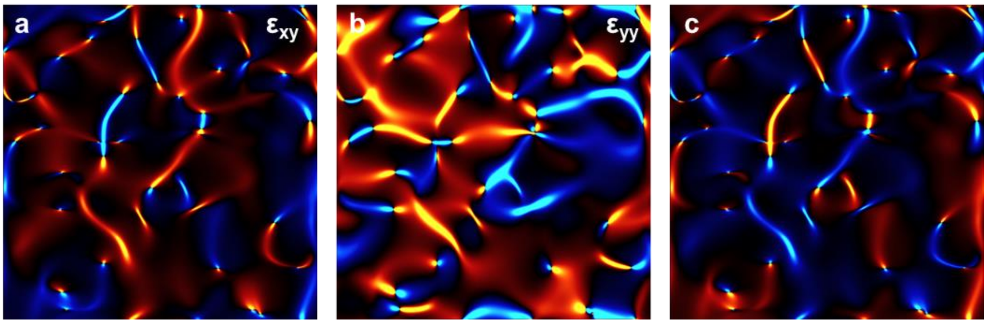

Fig. S7. Strain filed mapping by GPA with (a) ε_{xy}, (b) ε_{yy}, and (c) rotation.

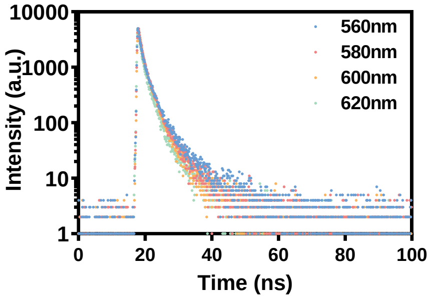

Fig. S8. Fluorescence decay curves of Pt-CDs aqueous solution. The excitation source was a picosecond pulse laser with a wavelength of 450 nm. The results showed the decay curves of Pt-CDs displayed approximated the lifetime.

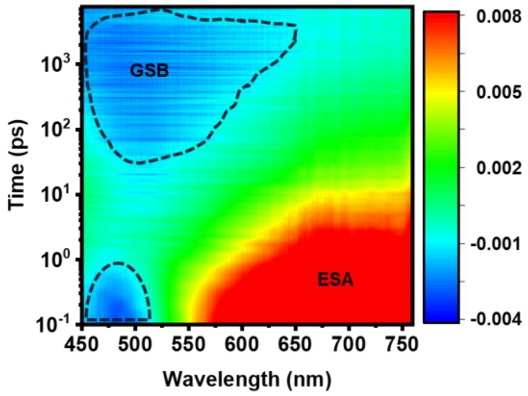

Fig. S9. 2D pseudo-color maps of the TA spectra of Pt-CDs treated with 1 min ultrasound with a pump wavelength of 400 nm. With the formation of J-type aggregates, one GSB signal around 480 nm was generated before 1 ps. Meanwhile, another GSB signal was also generated around 570 nm after 30 ps. During the decay process of the GSB signal at 480 nm, the GSB signal at 480 nm became stronger, suggesting a strong CT process from the monodisperse state to the J-aggregated state.

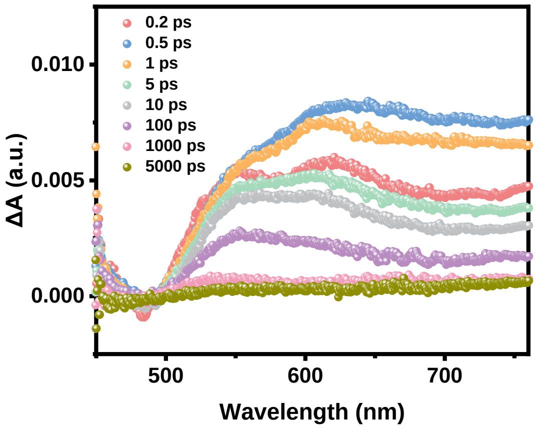

Fig. S10. TA spectra of Pt-CDs at indicated delay times. The results showed Pt-CDs exhibited a ground state bleaching (GSB) signal in 450-510 nm regions (GSB1), which can be attributed to the intrinsic (IC) state of the graphene-like plate.

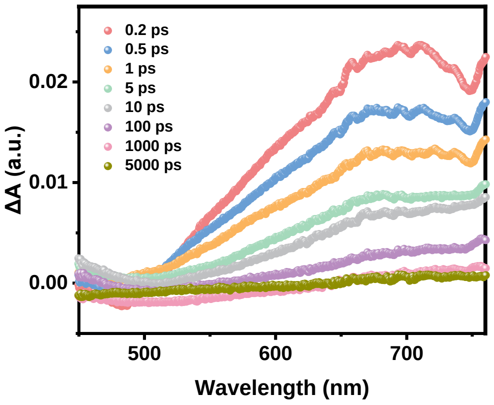

Fig. S11. TA spectra of Pt-CDs intermediates at indicated delay times. The results showed the GSB1 of Pt-CDs intermediates decayed gradually, while the GSB signals from 450 to 700 nm emerged.

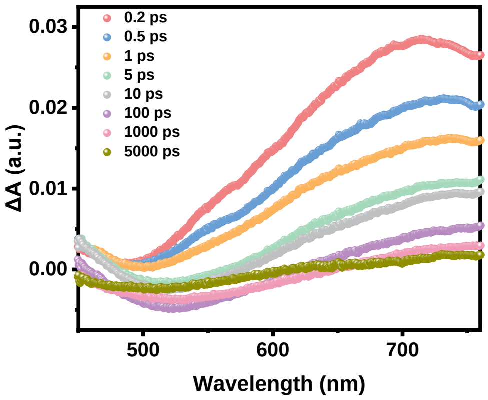

Fig. S12. TA spectra of HS-Pt-CDs at indicated delay times. The results showed the GSB1 signal of HS-Pt-CDs was much weaker and rapidly decayed, while broad and intense GSB signals covered from 450 to 700 nm (GSB2) emerged following the decay of the GSB1 signal.

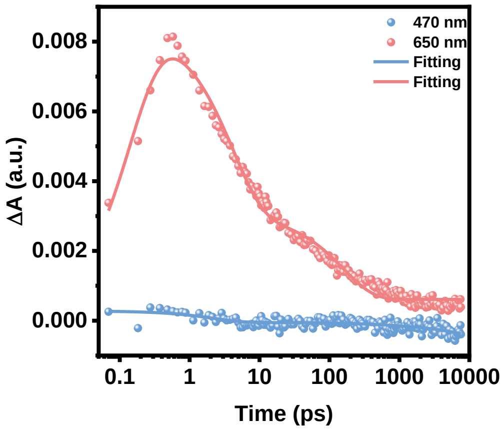

Fig. S13. TA kinetic traces of Pt-CDs collected at 470, and 650 nm at a pump wavelength of 400 nm. Dots are experimental points, and solid lines are fittings. The results showed there was little energy transfer (ET) happening from GSB1 to GSB2, indicating that the assembly among the Pt-CDs had not yet occurred.

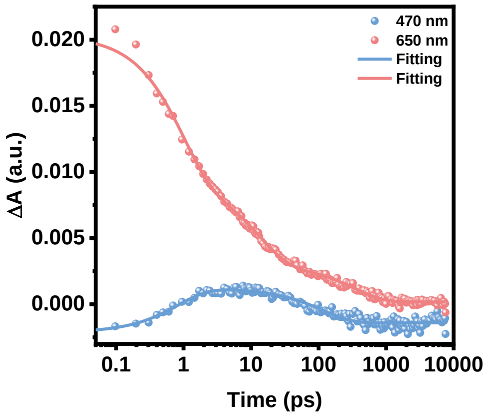

Fig. S14. TA kinetic traces of intermediates collected at 470, and 650 nm at a pump wavelength of 400 nm. Dots are experimental points, and solid lines are fittings. The results revealed the intermediates underwent a strongly rearranged electronic structures after assembly, with significant energy transfer (ET) happening from GSB1 to GSB2.

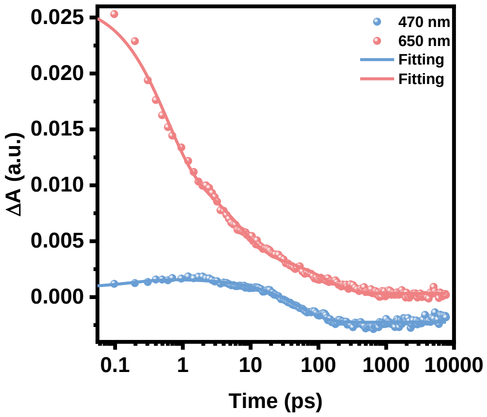

Fig. S15. TA kinetic traces of HS-Pt-CDs collected at 470, and 650 nm at a pump wavelength of 400 nm. Dots are experimental points, and solid lines are fittings. The results revealed the rearranged electronic structures gradually completed. The energy transfer (ET) happening from GSB1 to GSB2 was weakened.

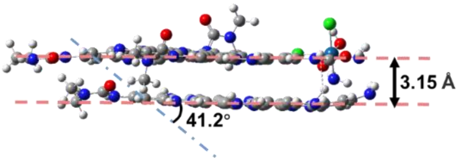

Fig. S16. J-type molecular packing diagrams of Pt-CDs in stick model.

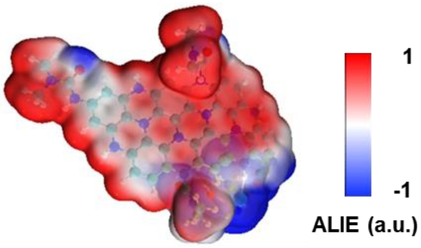

Fig. S17. Calculated electrostatic potential surface (EPS) distribution of Pt-CDs on the basis of the crystal structures at the B3LYP/6-31G(d) + LANL2DZ level. a negative electrostatic potential (red) indicates high electronic density, whereas a positive one (blue) corresponds to low electronic density. Apparently, the red regions were observed at the edge amide groups and central aromatic structure, indicating electron-rich domains. In contrast, the coordination with Pt caused strong positive charge localization in these regions. This pronounced charge asymmetry was likely to drive the molecular misalignment and slip-stacked packing arrangement.

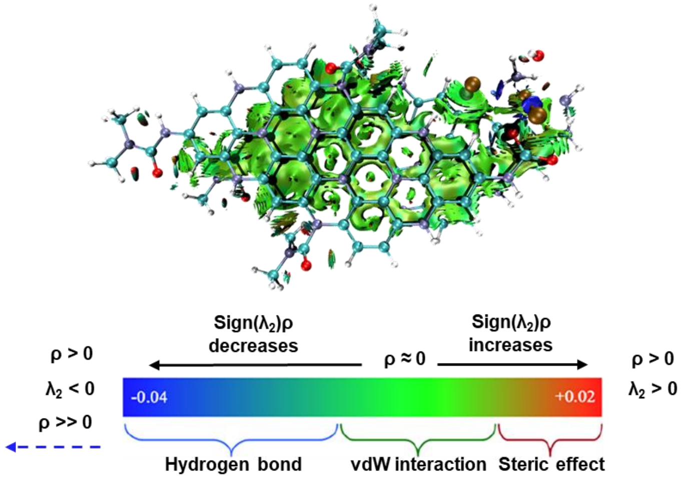

Fig. S18. Interaction region indicator (IRI) isosurface map (IRI = 1) of Pt-CDs dimer at the B3LYP/6-31G(d) + LANL2DZ level of theory. It was clear that the interaction between the Pt-CDs was mainly due to the van der Waals (vdW) interaction. Note that these vdW regions were predominantly localized between sp^2 conjugation domains.

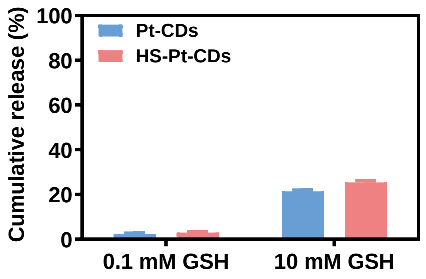

Fig. S19. Pt release amount in 24 h from 0.25 mg mL^{-1} Pt-CDs and HS-Pt-CDs aqueous solutions with 0.1 and 10 mM GSH.

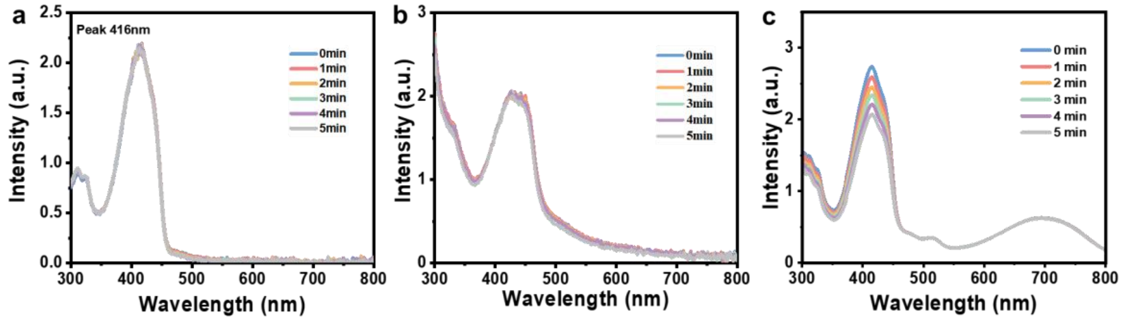

Fig. S20. ROS production of (a) oxoplain and (b) Pt-CDs by measurement of 1,3-Diphenylisobenzofuran (DPBF) peak at 416 nm upon different time 690 nm laser irradiation. (c) ROS production of HS-Pt-CDs by measurement of 1,3-Diphenylisobenzofuran (DPBF) peak at 416 nm upon different time 808 nm laser irradiation. The oxoplatin and Pt-CDs could not generate ROS upon the 690 nm light irradiation, as evidenced by the neglected downregulation of DPBF absorption at 416 nm. On the contrary, the HS-Pt-CDs also could be exited and produced amount of ROS under 808 nm light.

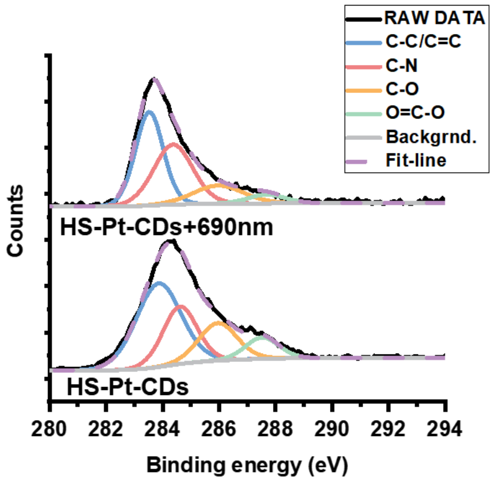

Fig. S21. High-resolution C1s spectra of HS-Pt-CDs and HS-Pt-CDs+690nm. The 690 nm laser was placed on the HS-Pt-CDs for 30 min (1.0 W cm^{-2}). As Pt(IV) moieties are linked on the carbon dots via O=C-O bond, they are easily broken upon the external stimuli. After NIR irradiation, the increasing O=C-O signal at 288 eV and C-O signal at 286 eV indicate Pt(IV) moieties were reduced and then released from the HS-Pt-CDs.

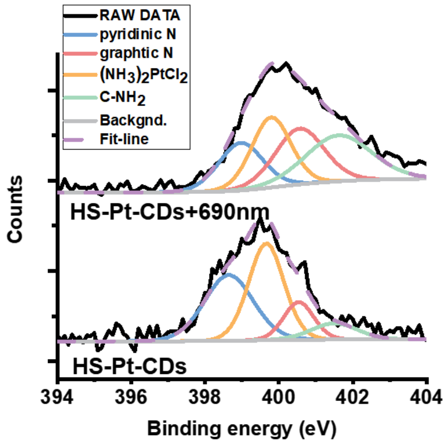

Fig. S22. High-resolution N1s spectra of Pt-CDs and Pt-CDs+690nm. The 690 nm laser was placed on the HS-Pt-CDs for 30 min (1.0 W cm^{-2}). The decreased $(NH_3)_2PtCl_2$ signal at 400eV and increased C-NH_2 signal at 402 eV after 690 nm light is in good agreement with the results of ^{1}H NMR, further demonstrating the photocatalysis of HS-Pt-CDs under NIR.

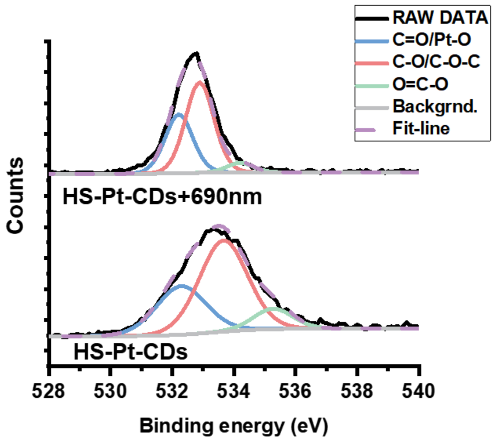

Fig. S23. High-resolution O1s spectra of HS-Pt-CDs and HS-Pt-CDs+690nm. The 690 nm laser was placed on the HS-Pt-CDs for 30 min (1.0 W cm^{-2}). As above mentioned, the decreased O=C-O signal at 535 eV and Pt-O signal at 532 eV after 690 nm light indicate the Pt(IV) moieties were reduced and then released from the HS-Pt-CDs.

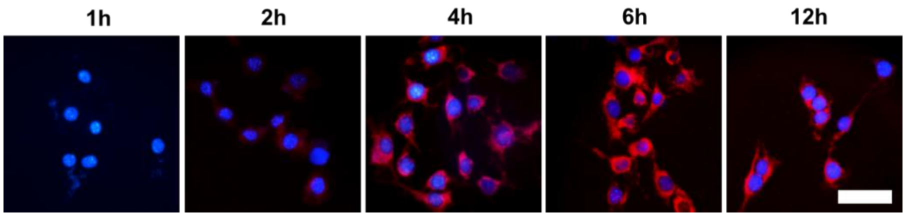

Fig. S24. Fluorescence images of EMT6 cells treated with HS-Pt-CDs at a Pt content of 20 μM. Scale bar = 20 μm.

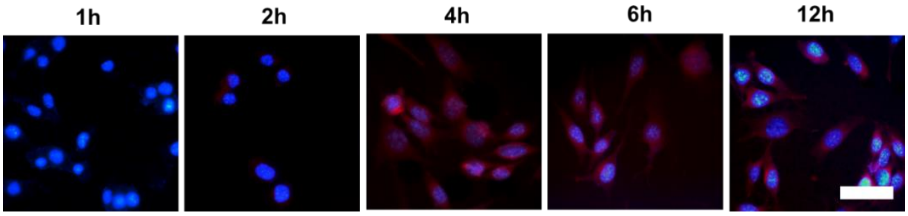

Fig. S25. Fluorescence images of EMT6 cells treated with Pt-CDs at a Pt content of 20 μM. Scale bar = 20 μm.

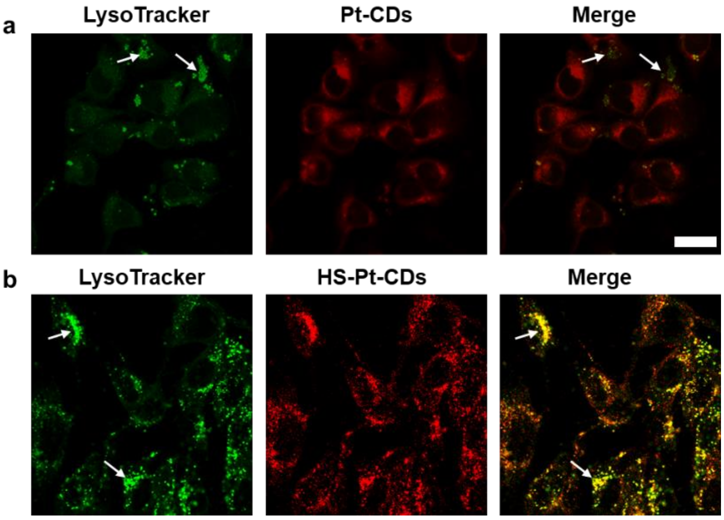

Fig. S26. Fluorescence images of EMT6 cells after culturing with (A) Pt-CDs and (B) HS-Pt-CDs for 3 h. Lysosome were stained by LysoTracker Green, respectively. Green: LysoTracker Green, Red: Pt-CDs or HS-Pt-CDs. (Scale bar: 10 μm). The results revealed the HS-Pt-CDs exhibited well colocalization with lysosomes after 3 h incubation, substantiating the cellular uptake of NPs by endocytosis and entrapment within endo/lysosomal compartments. Instead, Pt-CDs entered cells via simple diffusion, as shown the mismatch between the green and red signal.

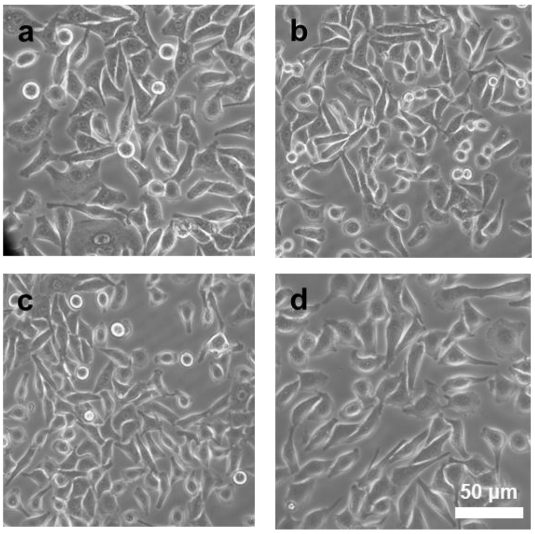

Fig. S27. Phase-contrast imaging assay of (a) cisplatin, (b) Pt-CDs, (c) cisplatin+Light, and (d) Pt-CDs+Light in EMT6 cells (Pt: 20 μM, NIR: 690 nm, 0.5 W cm^{-2}, 20 min).

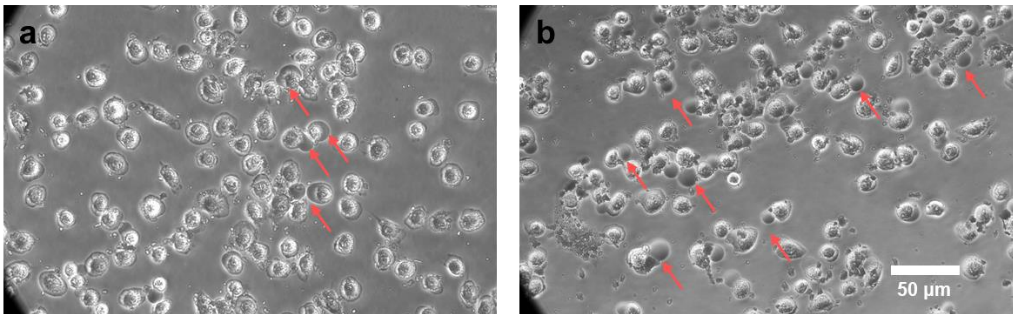

Fig. S28. Phase-contrast imaging assay of HS-Pt-CDs-induced pyroptosis (arrow) in EMT6 cancer cells (Pt: 20 μM, NIR: 690 nm, 0.5 W cm^{-2}, (a) 20 min and (b) 40 min).

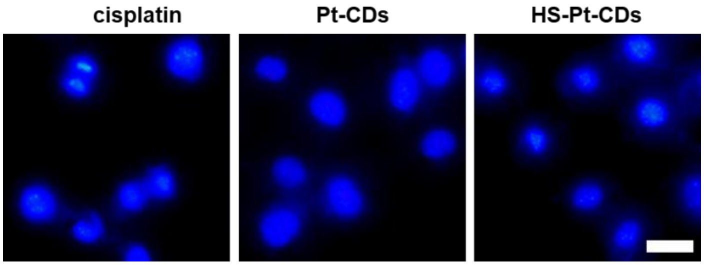

Fig. S29. CLSM images of surface-exposed CRT on EMT6 cells a treated with cisplatin, Pt-CDs, and HS-Pt-CDs at a Pt concentration of 20 μM. Scale bar = 20 μm. (690 nm 0.5 W cm^{-2}, 20 min).

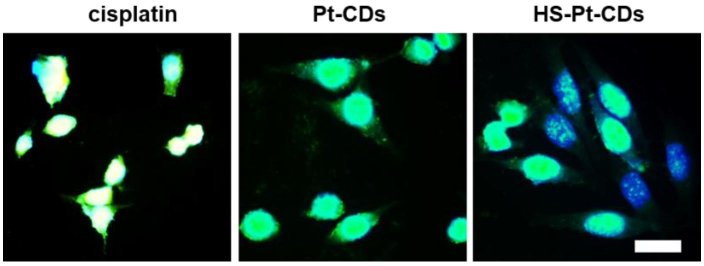

Fig. S30. CLSM images of HMGB1 release from EMT6 cells treated with cisplatin, Pt-CDs, and HS-Pt-CDs at a Pt concentration of 20 μM. Scale bar = 20 μm. (690 nm 0.5 W cm^{-2}, 20 min).

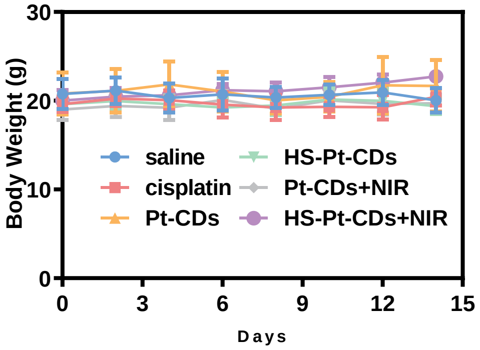

Fig. S31. Body weight variation of tumor-bearing mice during the treatment. Error bars indicate standard deviations (n = 5).

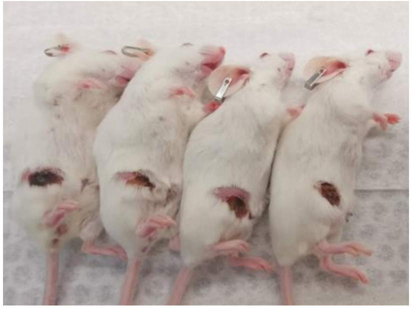

Fig. S32. Typical images of the EMT-6 tumor bearing mice treated with HS-Pt-CDs+690nm at Day 14. (690 nm, 1.0 W cm^{-2}, 20 min).

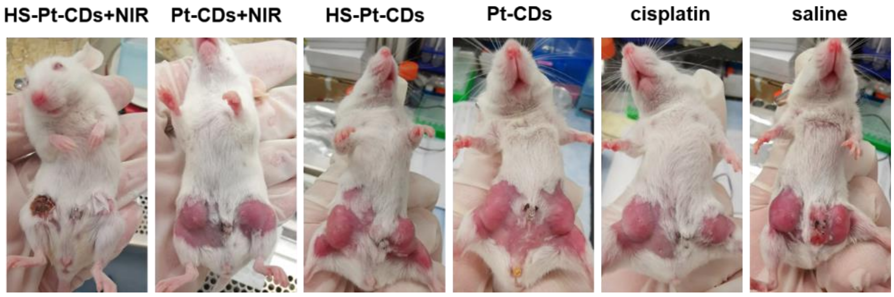

Fig. S33. Typical images of the EMT-6 tumor bearing mice treated with variable Pt-based formulations at Day 14.

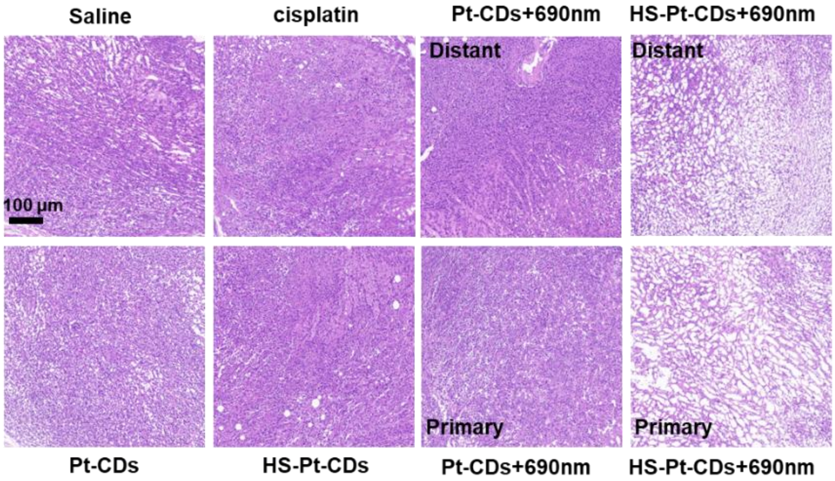

Fig. S34. H&E staining of the tumors after one round of treatment.

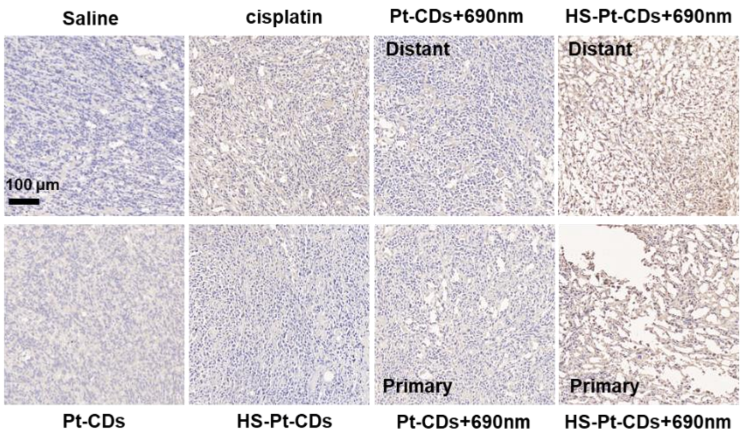

Fig. S35. Cleaved caspase 3 (c-Cas-3) staining of the tumors after one round of treatment.

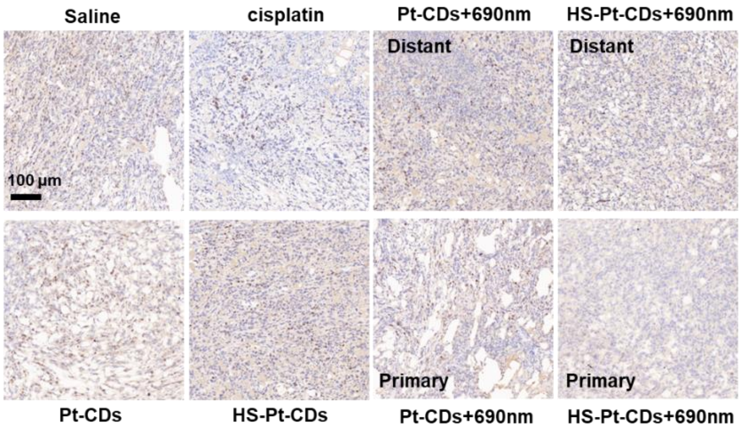

Fig. S36. The ki67 staining of the tumors after one round of treatment.

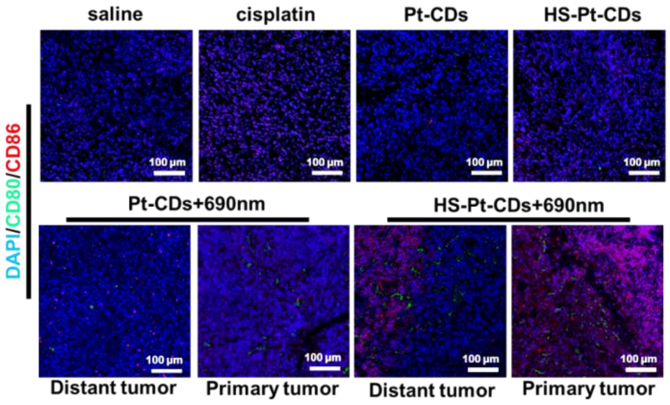

Fig. S37. The CD86 and CD80 staining of the tumors after one round of treatment. Red: CD86, Green: CD80, Blue: DAPI.

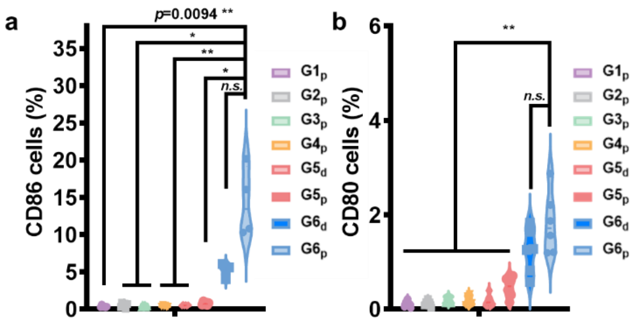

Fig. S38. Quantitative analysis of CD86 and CD80 cells in primary tumors and distant tumors from EMT-6 tumor bearing mice. The HS-Pt-CDs+690nm treatment recruited 14.35% and 1.82% of intratumoral infiltration of the CD86 and CD80 cells, respectively, representing 25-fold and 14.2-fold increase compared to cisplatin, respectively. Meanwhile, the distant tumors in the HS-Pt-CDs+690nm group also had high levels of CD86 and CD80 expression, measuring 6.8-fold and 2.7-fold higher than those of the Pt-CDs+690nm group.

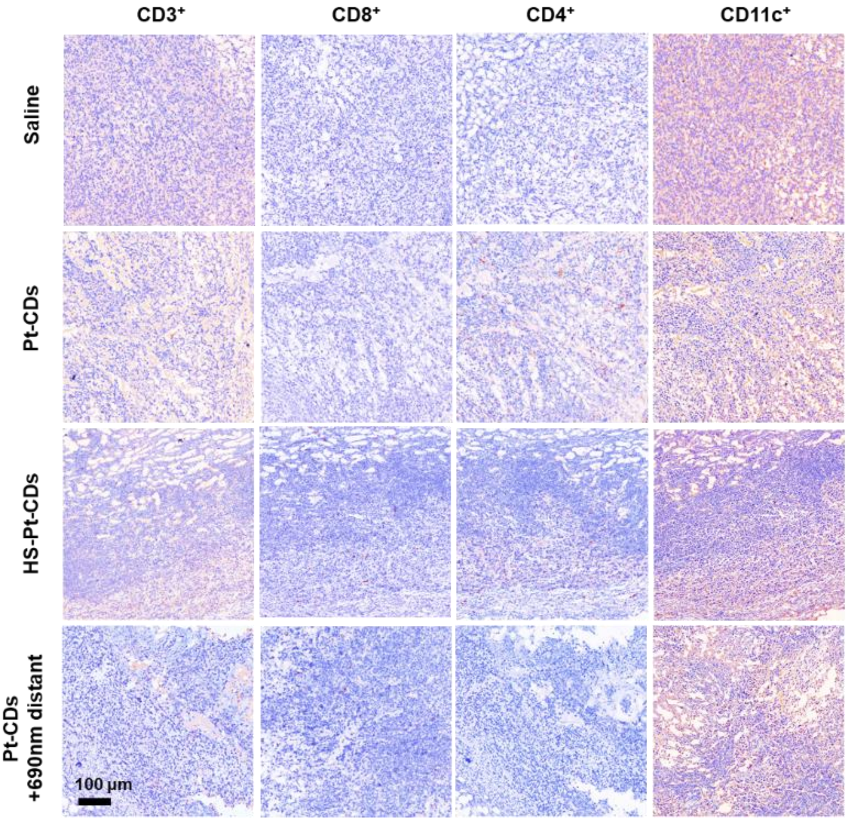

Fig. S39. $CD3^+$, $CD4^+$, $CD8^+$, and $CD11c^+$ immunohistochemical staining of primary tumors in Saline, Pt-CDs, and HS-Pt-CDs and distant tumor in Pt-CDs+690nm.

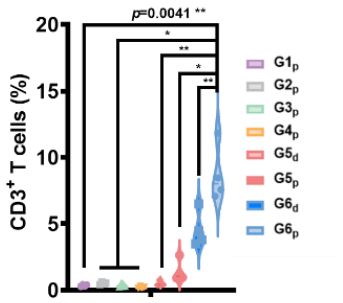

Fig. S40. Quantitative analysis of CD3⁺ T cells in primary tumors and distant tumors from EMT-6 tumor bearing mice.

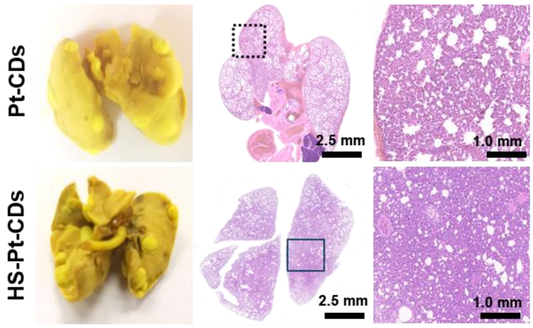

Fig. S41. Digital photographs of lungs with metastatic nodules stained by Bouin's fluid and H&E staining of lung sections from EMT6-tumor-bearing mice treated with Pt-CDs and HS-Pt-CDs.

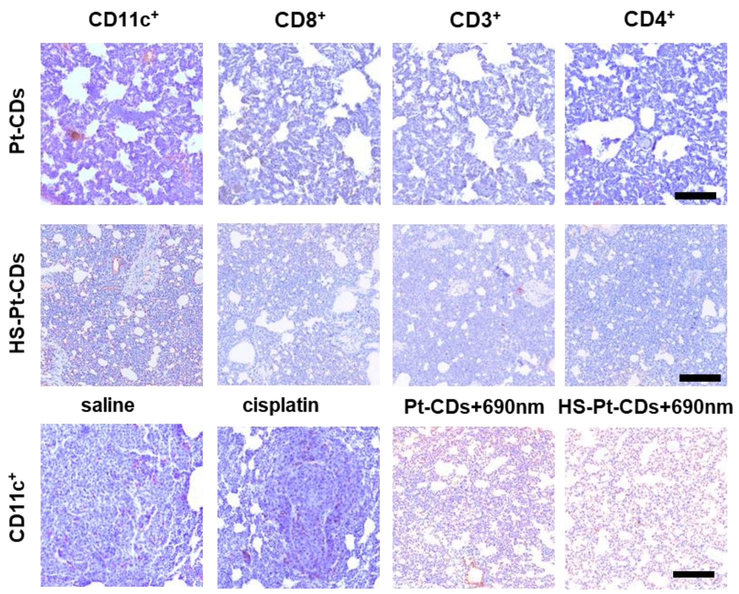

Fig. S42. CD11c⁺, CD3⁺, CD8⁺, and CD4c⁺ staining of the lungs from mice in each group. Scale bar: 50 μm.

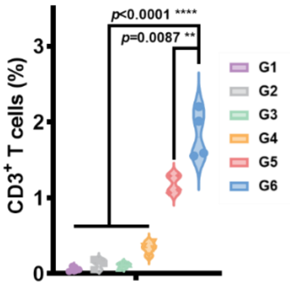

Fig. S43. Quantitative analysis of CD3+ T cells in lungs from EMT-6 tumor bearing mice. The HS-Pt-CDs+690nm treatment recruited 1.84% of intratumoral infiltration of the CD3+ T cells, representing 13.5-fold and 1.6-fold and increase compared to cisplatin and Pt-CDs+690 nm, respectively.

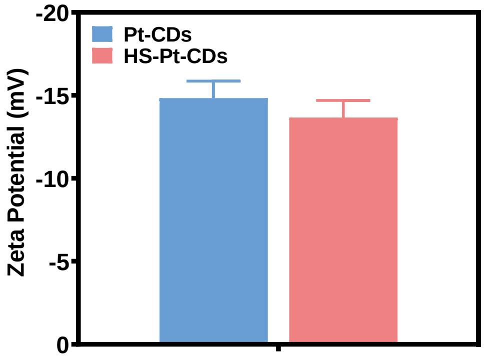

Fig. S44. Zeta potential of Pt-CDs and HS-Pt-CDs in aqueous solutions. The zeta potential of Pt-CDs and HS-Pt-CDs were -14.8 mV and -13.6 mV.

References

1. Hytch, M. et al. Quantitative measurement of displacement and strain fields from HREM micrographs. *Ultramicroscopy* **74**, 131–146 (1998).

2. W. Humphrey, et al. VMD: visual molecular dynamics. *Journal of molecular graphics* **14**, 33-38 (1996).

3. L. Goerigk, et al. Efficient and Accurate Double-Hybrid-Meta-GGA Density Functionals-Evaluation with the Extended GMTKN30 Database for General Main Group Thermochemistry, Kinetics, and Noncovalent Interactions. *Journal of chemical theory and computation* **7**, 291-309 (2010).
